# Enhancement strategies for transdermal drug delivery systems: current trends and applications

**DOI:** 10.1007/s13346-021-00909-6

**Published:** 2021-01-20

**Authors:** Delly Ramadon, Maeliosa T. C. McCrudden, Aaron J. Courtenay, Ryan F. Donnelly

**Affiliations:** 1 School of Pharmacy, Medical Biology Centre, Queen’s University Belfast, 97 Lisburn Road, Belfast, BT9 7BL UK; 2 Faculty of Pharmacy, Universitas Indonesia, Depok, Indonesia; 3 School of Medicine, Dentistry and Biomedical Sciences, Whitla Medical Building, Queen’s University Belfast, 97 Lisburn Road, Belfast, BT9 7BL UK; 4 School of Pharmacy and Pharmaceutical Sciences, Ulster University, Coleraine, BT52 1SA UK

**Keywords:** Active, Enhancement methods, Microneedles, Passive, Skin, Transdermal drug delivery

## Abstract

Transdermal drug delivery systems have become an intriguing research topic in pharmaceutical technology area and one of the most frequently developed pharmaceutical products in global market. The use of these systems can overcome associated drawbacks of other delivery routes, such as oral and parenteral. The authors will review current trends, and future applications of transdermal technologies, with specific focus on providing a comprehensive understanding of transdermal drug delivery systems and enhancement strategies. This article will initially discuss each transdermal enhancement method used in the development of first-generation transdermal products. These methods include drug/vehicle interactions, vesicles and particles, *stratum corneum* modification, energy-driven methods and *stratum corneum* bypassing techniques. Through suitable design and implementation of active *stratum corneum* bypassing methods, notably microneedle technology, transdermal delivery systems have been shown to deliver both low and high molecular weight drugs. Microneedle technology platforms have proven themselves to be more versatile than other transdermal systems with opportunities for intradermal delivery of drugs/biotherapeutics and therapeutic drug monitoring. These have shown that microneedles have been a prospective strategy for improving transdermal delivery systems.

**Graphical abstract:**

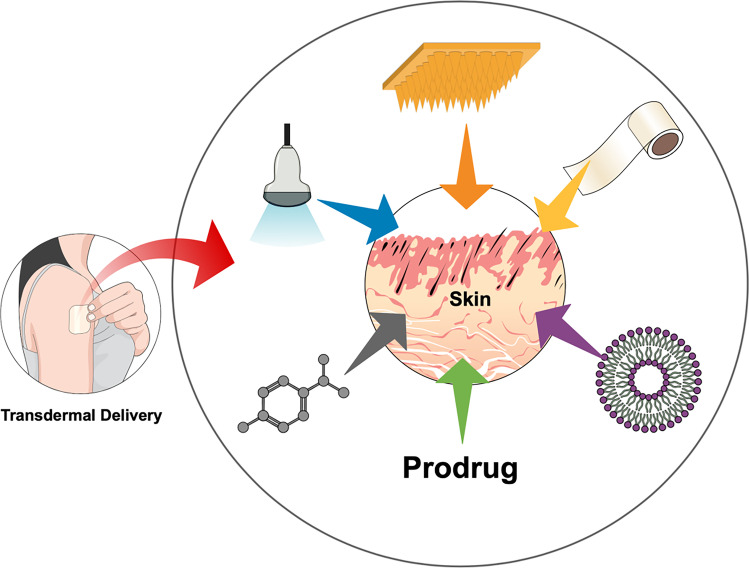

## Introduction

### Transdermal drug delivery systems

Innovation in drug delivery systems is a key strategy employed to improve the bioavailability of active pharmaceutical ingredients (APIs). To date, oral delivery systems remain the most preferable method for administrating API due to the benefits offered, such as dosage form variety, painless ease of administration, convenience, self-administration, high safety, and patient compliance [1]. Despite these advantages, oral delivery systems have some limitations such as poor drug stability in the gastrointestinal tract and subjection to first pass metabolism. For instance, there is a possibility of drug degradation caused by enzymatic reaction or exposure to the acidic environment in the stomach [2]. Moreover, the solubility issues of drugs in the intestinal fluid and their permeability through the intestinal membrane may act as rate limiting steps in drug absorption, causing low bioavailability [3]. These drawbacks are routinely observed in the delivery of peptide or protein-based drugs [2]. As a result, intravenous (IV) injection is designated as one of the most promising delivery system for proteinaceous drugs, as it can achieve up to 100% bioavailability, accurate dosing and hepatic metabolism avoidance [4]. It is not surprising, however, that the IV administration route has some potential disadvantages, for example, it is an invasive delivery method, causing pain, low patient compliance, and sharps waste disposal considerations add significant costs [1, 5]. With a view to potentially overcoming some of these disadvantages, the transdermal route has been explored as another prospective route for enhancing delivery of peptide drugs [6].

**Fig. 1:**
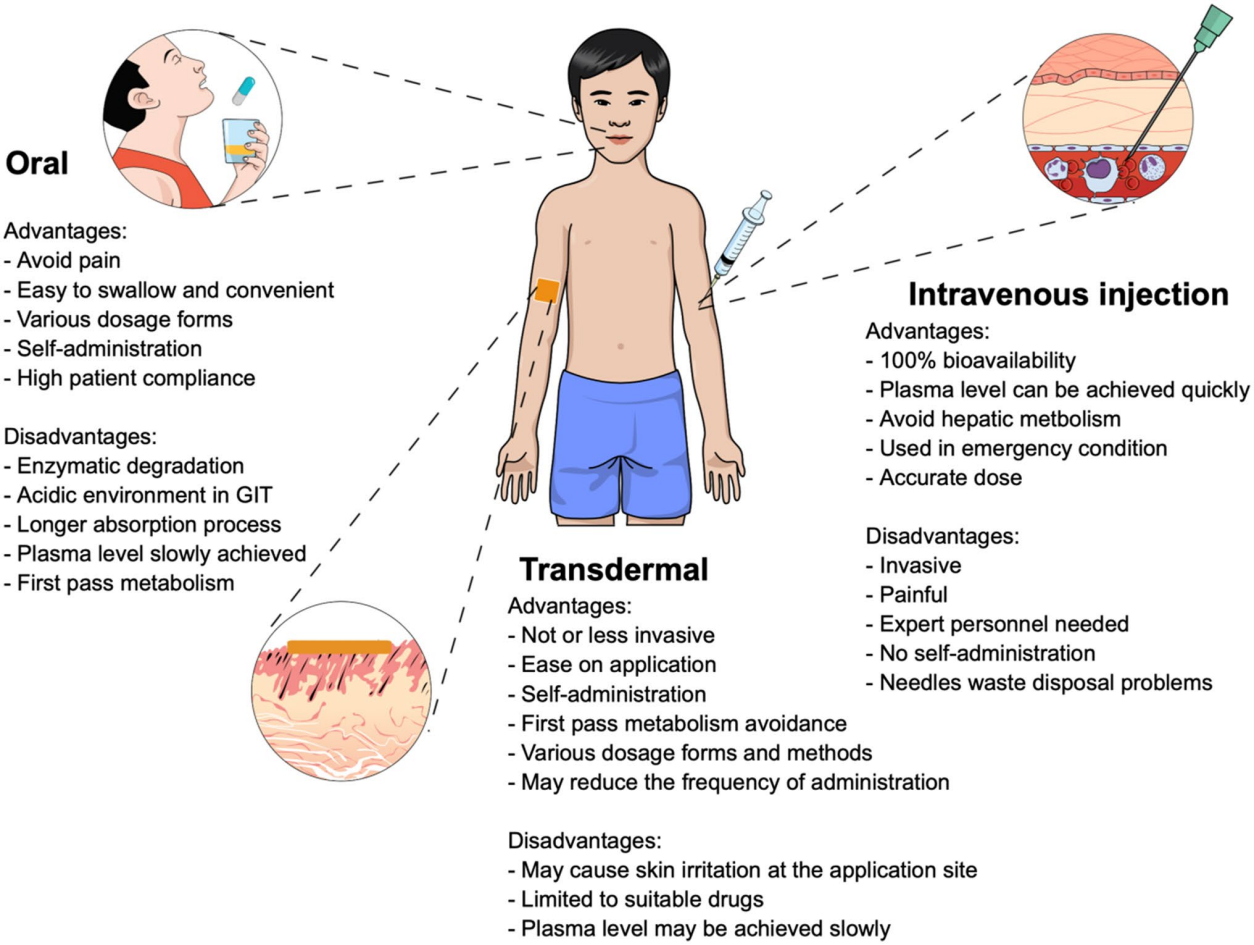
Comparison of three different routes of drug administration: oral, intravenous injection and transdermal

Transdermal drug delivery systems use the skin as the drug administration site [7]. The administered drug is absorbed into the systemic circulation via blood vessels in the skin and then circulates around the body [8]. Transdermal drug delivery systems offer some advantages for patients, such as being less invasive (some methods are entirely noninvasive), first-pass metabolism avoidance, ease of application and administration, no need for expert personnel, and the potential to reduce frequency of administration [9, 10]. Additionally, this technology has been used for the delivery of different varieties of drugs, both hydrophilic and hydrophobic compounds. The benefits documented above have garnered interest from pharmaceutical researchers to develop and explore transdermal drug delivery systems, particularly in modifying or breaching the *stratum corneum* to enhance drug permeation through the skin. A comparison of the three different routes of drug administration detailed herein is summarised in Fig. 1.

### Skin

**Fig. 2:**
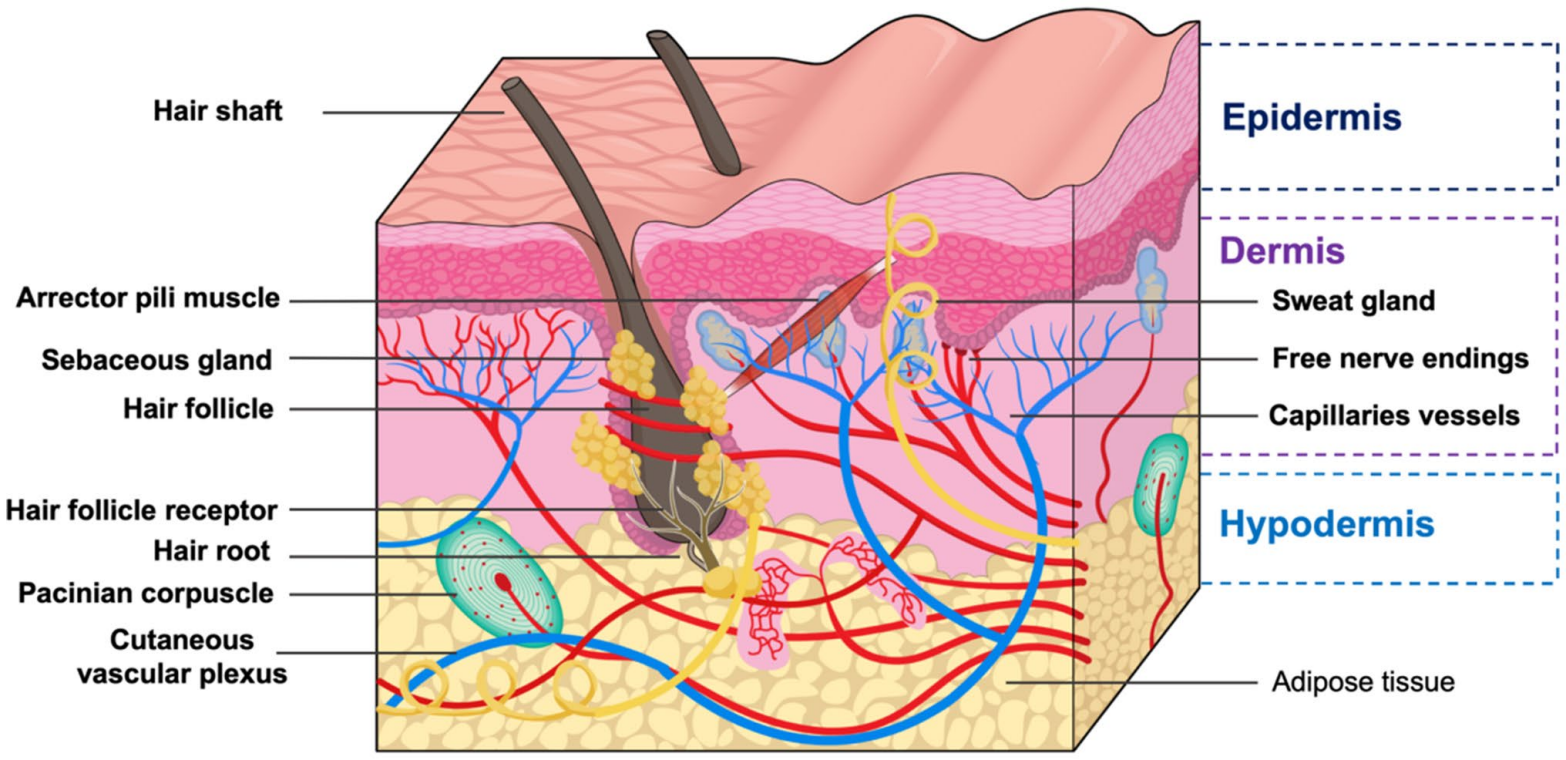
Schematic illustration of the anatomy of the skin

Skin is the first line of protection for the body from the external environment. It has an area of approximately 1.5–2.0 m^2^ and accounts for 15% of the total body mass of an adult person [11]. Skin, as the largest organ, functions to protect the body from external disturbances, including physical, mechanical and chemical assault [12]. Moreover, due to the abundance of melanin, the skin also protects the human body from ultraviolet (UV) radiation from the sun [13]. Another important function of the skin is the maintenance of homeostasis via the thermoregulation system [14]. Sweating is one such thermoregulation mechanism performed by human skin [15]. Skin is also responsible for the excretion of several substances, such as xenobiotics, excessive lipids, sodium chloride, urea, uric acid, ammonia and lipid [16, 17]. In addition, researchers have been using the skin as the main absorption site for various kinds of drugs, both for local and systemic delivery as a consequence of the many blood capillaries residing in the dermis [7]. A detailed anatomy of the skin is presented in Fig. 2. The outermost layer of human skin is the epidermis, approximately 50–100 µm, dependent on where it is on the body [18].

**Fig. 3:**
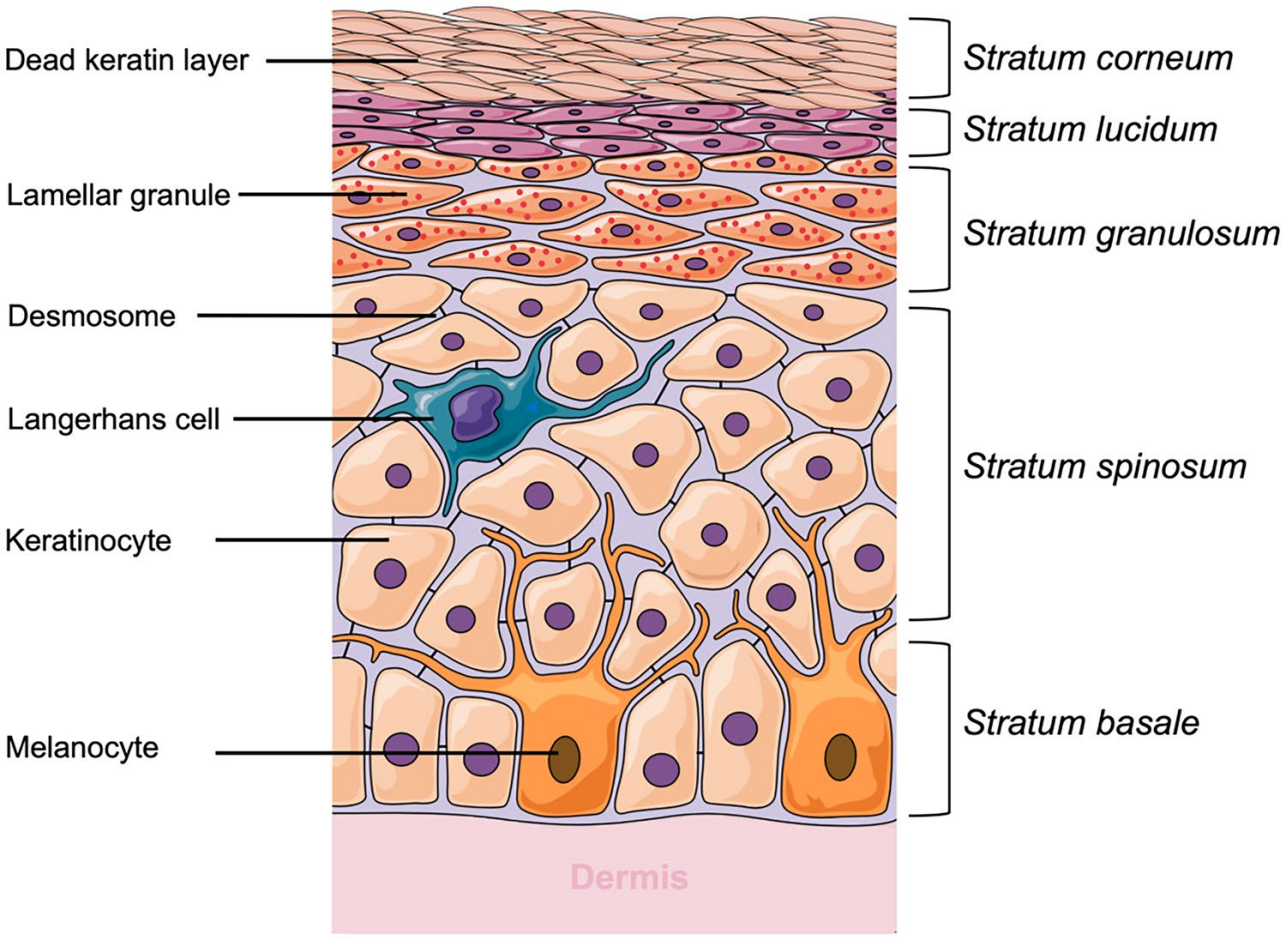
Schematic representation of epidermis layer of human skin

Specifically, the epidermis consists of five different layers, illustrated in Fig. 3, that function in the mechanism of skin regeneration. The *stratum corneum* (SC), the most superficial layer of the epidermis, has a thickness of 10–20 µm, consisting of 15–30 corneocyte cell layers. This layer regenerates every 4 weeks [19, 20]. The SC is made up of keratin proteins that comes from dead keratinocyte cells in the deeper layers, in a process termed cornification and, hence it is also known as a ‘horny layer’ [21]. Furthermore, SC is also composed of lipids, such as ceramides (30–40%) [22, 23], cholesterols, cholesterols esters, free fatty acids, squalene, wax esters and triglycerides [12, 24]. Below the SC, there is a clear and thin layer of skin, namely *stratum lucidum.* The *stratum lucidum* consists of 2–3 layers of keratinocyte cells and is found only in digits, palms and soles [25]. The dead corneocytes of the SC are brought up from this layer.

The next layer under the *stratum lucidum* is the *stratum granulosum*. In this layer, the cells have a thicker membrane compared to the first two layers. The granulosum originates from granules within the living cells which are formed by the accumulation of keratohyalin, a protein structure found in the granules [26]. The *stratum spinosum*, an epidermal layer under the *stratum granulosum*, consists of 8–10 layers of keratinocytes [25]. The presence of cell connectors, namely desmosomes, between the cells causes this layer to be called the ‘spiny’ layer (*stratum spinosum*) [27]. Antigen presenting cells, known as Langerhans cells, are found in this layer [28]. These dendritic cells have a responsibility to engulf bacteria or exogenous particles and damaged cells by phagocytosis [29]. Finally, the deepest layer of the epidermal skin is the *stratum basale* which directly contacts the dermis via interconnecting collagen fibers. In the *stratum basale*, cells proliferate and become primary cells for keratinocytes that are present in the upper epidermal layers [30]. Merkel cell, a functional cell of the sensory systems, and melanocytes are found in this layer [31, 32].

Underlying the epidermis, the second layer of skin in the integumentary system is the dermis. There are two layers in the dermis, termed the papillary and reticular layers [25]. Specifically, adipocytes, blood vessels and lymphatic capillaries are found in the papillary layer of the dermis [33]. The reticular layer is much denser than the papillary layer, due to the high content of collagen fibers [34], affording it elasticity for functioning in movement. The dermal layer plays a key role in immune function, due to the presence of phagocytes, fibroblasts, leucocytes and mast cells [35]. Moreover, there are an abundance of hair follicles, sebaceous and sweat glands in the dermis, as a consequence of its role in sweating and sebum secretion mechanisms [36].

Underlying the dermis is the deepest skin layer, the hypodermis. The hypodermis, also known as the subcutaneous layer or the superficial fascia, functions as a connecting tissue between skin, muscle and bones and as a result this layer is rich in proteoglycans and glycosaminoglycans [37]. Furthermore, an abundance of adipose tissue in the hypodermis provides thermal insulation to keep the body warm [38].

### Drug absorption via the skin

The skin is a potential site for drug absorption, due to the large surface area of this organ [39]. Following the application of drug-containing dosage forms onto the skin, drug will be released into the skin. However, the absorption of drug through the skin is very challenging, because there is the first barrier that has to be passed, the SC [8]. Structurally, the SC is composed of dead keratinocytes which, together the ceramide lipid component, form a dense structure which is known as a ‘brick-and-mortar’ arrangement [40, 41]. The ‘brick’ component of the SC is keratin, an acidic or basic to neutral protein product of keratinocytes, while the ‘mortar’ is comprised of lipids. The keratinocytes are connected to each other by glycoprotein desmosomes, termed corneodesmosomes [42]. In order for administered drugs to be absorbed into the circulation, they must first permeate into the skin via this molecular architecture.

**Fig. 4:**
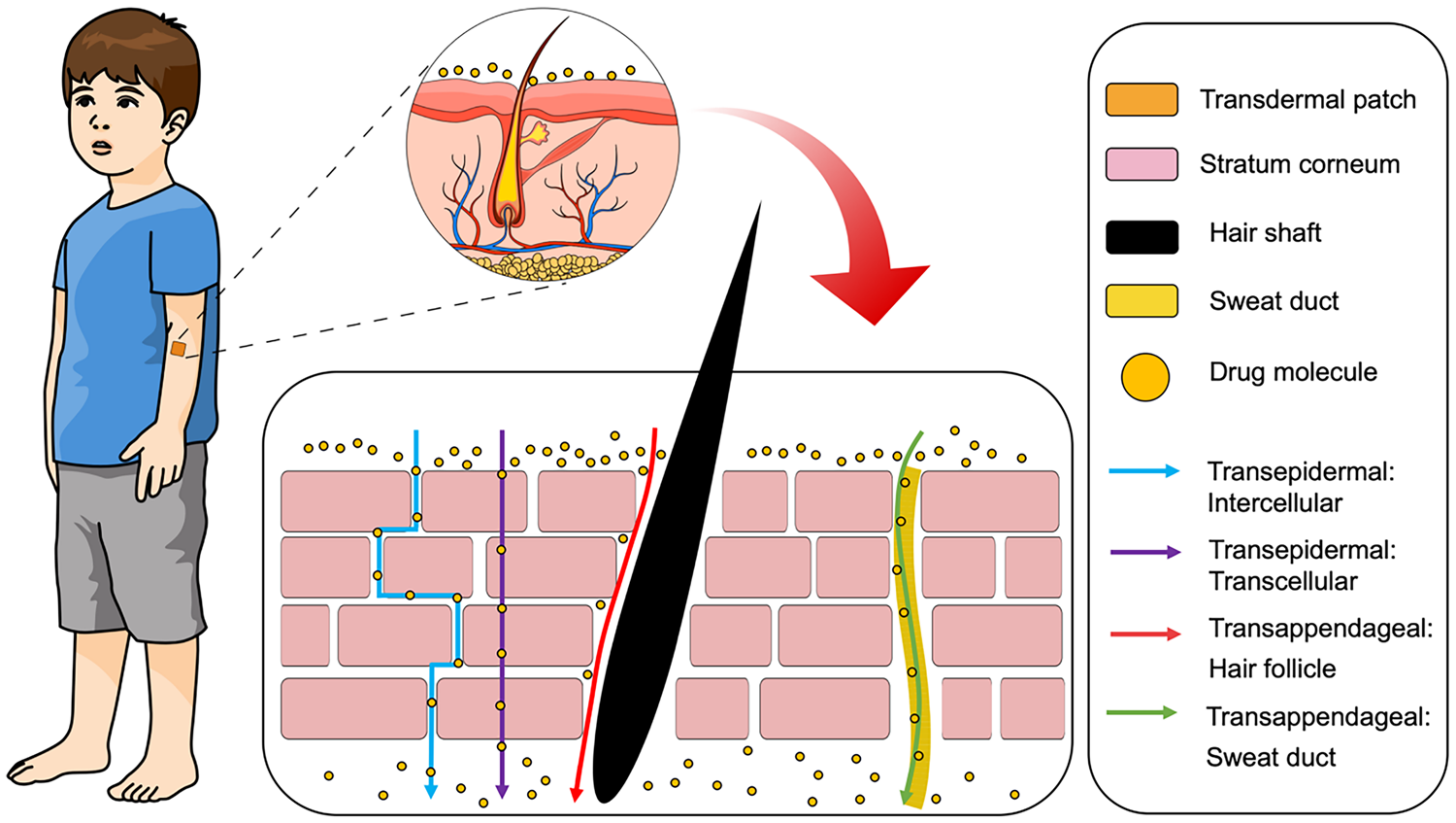
Schematic representation of transdermal drug delivery mechanisms

Generally, drug absorption from the skin via the SC can be distinguished into two pathways, transepidermal and transappendageal, as depicted in Fig. 4. The first pathway and the main absorption route is known as transepidermal [43]. The large surface area of the SC allows drug from transdermal patch to spread onto the skin surface and permeate into the cells (transcellular) [44] or interspaces between the cells (intercellular) [45]. The transepidermal route can be further subdivided into two pathways, namely transcellular and intercellular. In the transcellular route, drugs diffuse through SC cells during the absorption process. Therefore, drugs have to pass the membranes, which are composed of lipid bilayers [39]. This route is mostly taken by hydrophobic drugs because of the hydrophobic properties of lipid complex in the cell membranes of the SC [43]. The second route is the intercellular, in which the drugs have to diffuse through the lipid matrix of the intercellular space of residing keratinocytes in the SC [46]. Hydrophilic compounds or small molecules are transported via this route to reach vascular capillaries in the dermis [47]. The intercellular route is the dominant pathway for drug absorption and is primarily dependent on a specific balance of the drug molecule to be both sufficiently lipid and aqueous soluble. [48].

The second pathway of drug absorption from the skin is transappendageal [49] which is defined as drug delivery via hair follicles or sweat glands in the skin [50]. This route is necessary for the transport of polar or ionisable compounds and is useful for transport of large macromolecules which have problems passing through the epidermal cells due to the molecular size and different partition properties [43]. Nevertheless, the usage of this pathway is somewhat limited due to the smaller absorption area (~ 0.1% of total skin area), compared to that available for the transepidermal route [7]. Thus, researchers have developed methods to enhance drug absorption across the skin by modifying the structure of the SC, either chemically, physically or using combinations of these methods. In the following sections, the development of transdermal products and several technologies for enhancing drug absorption via the skin is discussed.

## A brief history of transdermal products

The use of the skin for delivery of various kinds of compounds has been widely explored over many centuries [51]. The ancient populations in Africa administered different types of traditional plants and minerals topically for cosmetic purposes or treatment of skin diseases [52]. For example, in 4000 BC, ancient Egyptians had discovered the use of natural resources, such as henna, red ochers and kohl, for skin care and cosmetics [53]. In 1500 BC, they wrote hundreds of drugs and prescriptions on a papyrus paper, namely *Ebers Papyrus* (manuscript on medicine) [54]. One example of the information written in this book was the use of the tiger nut for covering skin wounds [54].

Some thousand years later, Galen, a Greek physician, introduced the first cold cream containing an emulsion of vegetable oil, beeswax and water for skin treatment [55, 56]. They used the cold cream for skin wounds, burns and joint pains, due to its perceived antimicrobial activity [57]. This invention was followed by the utilisation of bandages and plasters by ancient Chinese populations for administrating herbal mixtures [51]. They mixed herbal ingredients with natural rubber gums and applied the plaster to the skin for localised treatment. One of primary transdermal formulations found in the fifteenth century was *Unguentum Hydrargyri*, an ointment formulation containing mercury for treatment of syphilis [58, 59]. In 1880, a plaster-based formulation (‘gutta-percha plaster gauze’) was developed by a German pharmacist, Paul Carl Beiersdorf, to treat skin disorders [60]. One of the most well-known plasters was *Emplastrum belladonnae* made of *Atropa belladonna* leaves for treatment of tuberculosis and tumours [51]. However, it was not always fully believed that drugs could be delivered into the circulation.

**Fig. 5:**
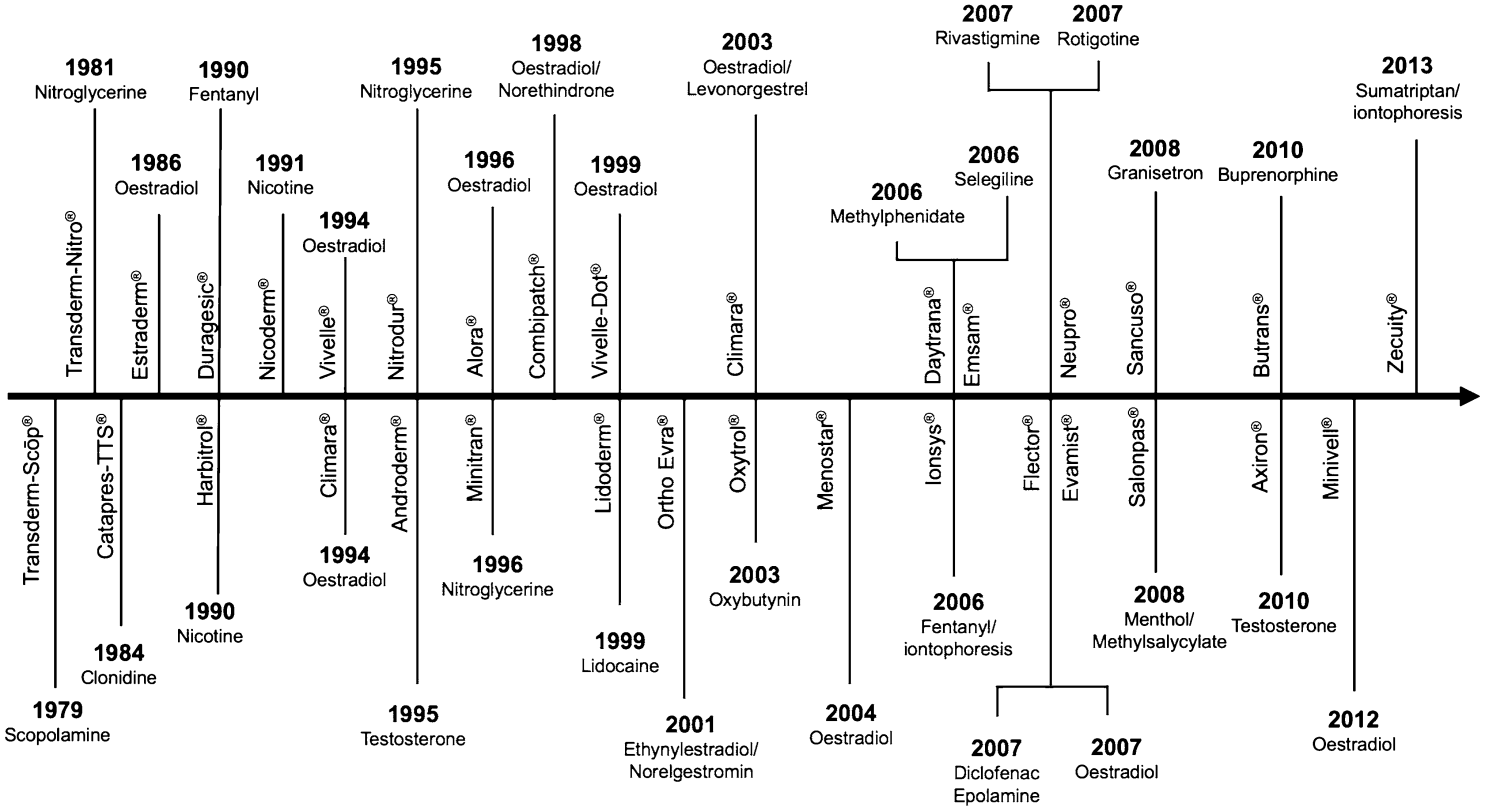
A timeline illustrating the journey of marketed transdermal products from 1981 until 2013

Then, in the twentieth century, some incidents of accidental intoxication were observed, for example poisoning by spills of phenol on the skin [61]. This phenomenon gave significant insights into the understanding of topical and transdermal drug delivery systems. As a result, in the 1950s, the first transdermal product in the form of an ointment was released to treat angina pectoris, namely Nitrol^®^ (2% nitroglycerin ointment) [51]. Nevertheless, this product had limitations, in terms of the application (greasy and not reproducible) and the frequency of administration (several times a day). Therefore, scientists were incentivised to develop ‘measured-dose’ transdermal delivery systems for different drugs to reduce the frequency of administration. Figure 5 presents a summary of marketed transdermal products, based on the available data in previous papers [10, 51, 62–64].

The first transdermal product containing scopolamine (Transderm Scōp^®^) was marketed in 1979. This product was used over 3 days for the treatment of motion sickness at sea. The development of Transderm Scōp^®^ had proven that transdermal delivery of scopolamine could reduce some of the side effects of this drug, when compared to oral administration. Consequently, some other APIs were formulated into transdermal dosage forms (Fig. 5). Following the scopolamine-containing product, Catapress-TTS^®^, a clonidine-loaded transdermal patch, was released in 1984 to treat hypertension. Additional transdermal products were also developed and marketed in 1986 (Estraderm^®^) and 1990 (Harbitrol^®^ and Duragesic^®^). From 1991 until 2004, marketed transdermal products were dominated by hormone-containing contraceptives, such as oestradiol, testosterone, ethynyl estradiol, norelgestromine and levonorgestrel. This suggested that at the beginning, transdermal products were intended predominantly for the delivery of hydrophobic drugs, composed of sterols [65].

From 2005 until 2013, several different types of drugs were also formulated into transdermal products, such as selegiline (Emsam^®^), methylphenidate (Daytrana^®^), fentanyl (Ionsys^®^), diclofenac epolamine (Flector^®^), a combination of menthol/methylsalycylate (Salonpas^®^) and sumatriptan (Zecuity^®^). Specifically, Ionsys^®^ and Zecuity^®^ are examples of transdermal product which coupled with iontophoresis for enhancing drug absorption from transdermal patch. Recently, some transdermal products, such as Secuado^®^ (asenapine for schizophrenia) and Twirla^®^ (ethinyl estradiol and levonorgestrel), were approved by FDA in 2019 and 2020, respectively [66]. The development of each transdermal product has been predeceased with advances in knowledge and understanding of transdermal drug delivery, resulting today, in a wide range of transdermal products available to patients and clinicians. To ensure progression it is imperative to understand a range of technologies that may be useful for enhancing drug absorption via the skin.

## Technologies for enhancing transdermal delivery

Transdermal drug delivery offers some benefits when compared to the other administration routes, such as first-pass metabolism avoidance and ease of self-administration. However, due to the dense cellular architecture and the hydrophobic characteristics of the SC, not all drugs are eligible to be administered using a conventional transdermal delivery system. There are several factors that may affect drug absorption into the skin.

The first factor affecting skin absorption is the physiology of the skin. For instance, the thickness of the SC and the amount of lipid in different parts of the skin layers, where the transdermal patch is applied, may influence the absorption rate of drugs into the skin [8]. The quantity of capillary blood vessels in certain skin body parts may have an impact on the rate of drug absorption into the circulation [67]. Moreover, the presence of hair follicles and sweat ducts may also contribute to a greater amount of drug permeating into the body, as a consequence of transfollicular drug delivery [50]. Body temperature affects the vasodilatation of skin capillaries and blood flow, resulting in higher rates of absorption [68, 69]. Furthermore, a higher amount of drug permeation may be achieved using an occlusive system to over-hydrate the skin [70].

Since the SC is composed of nonpolar lipid and neutral keratin proteins, drugs have to possess sufficient solubility both in water and oil to be absorbed into the skin [48]. In other words, the log partition coefficient (Log P) of the drug should be in the range of 1.0–3.0 [71]. Conventional transdermal products utilise a passive diffusion of drugs upon permeation into the skin. Optimal drug absorption can be achieved when the molecular size of the drug compound is less than 600 Da [43].

In terms of chemical factors, the degree of ionisation of the drug has a significant impact on its absorption through the skin. For example, unionised compounds may have greater drug permeation when compared to the ionisable drug, due to the hydrophobic similarity with the SC [48]. The melting point of the drug may also affect its permeation into the skin. When a drug has a low melting point, its solubility in the SC is higher, potentially resulting in a greater amount of drug permeating into the skin [72].

**Fig. 6:**
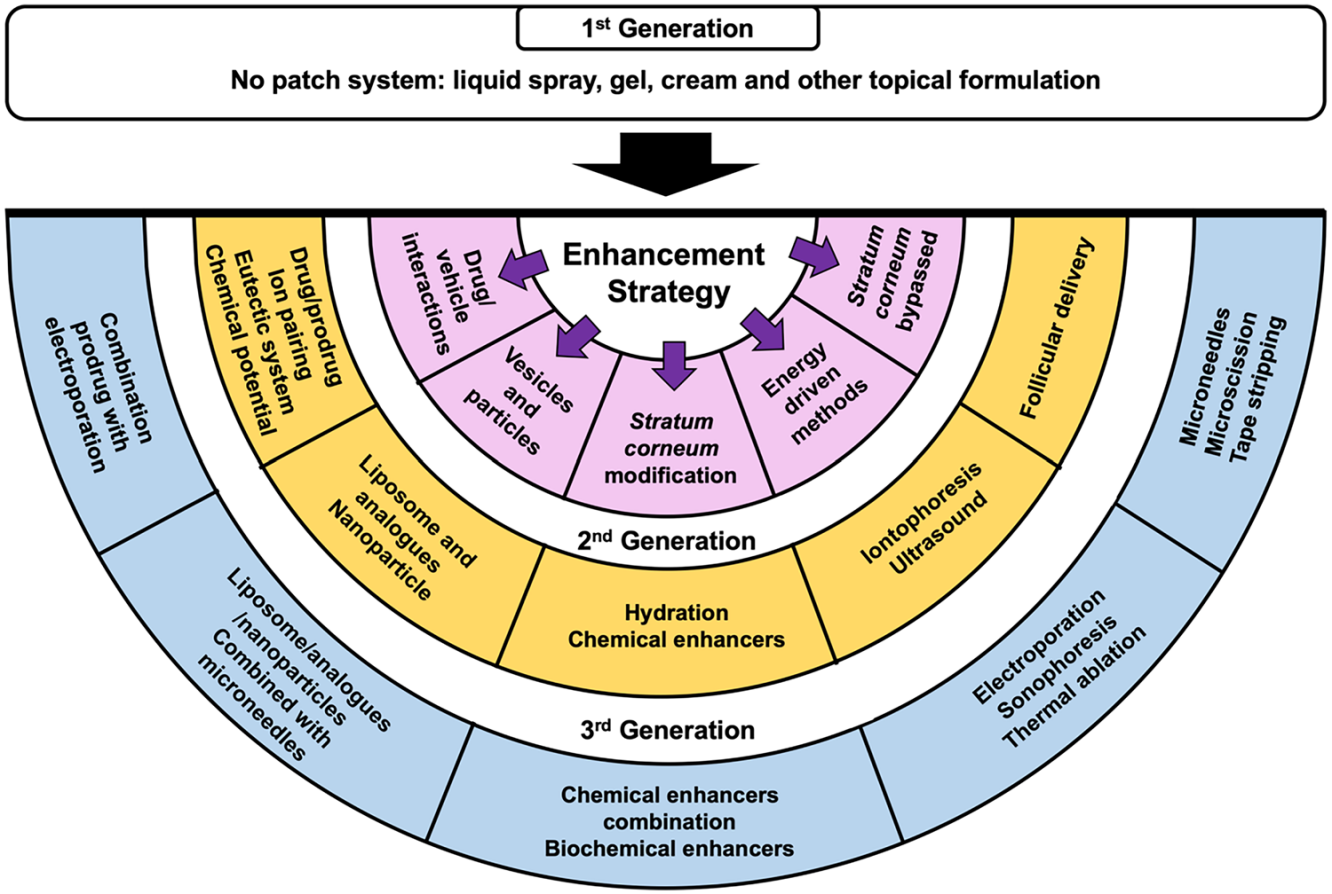
Summary of the technologies utilised for enhancing transdermal drug delivery

Taking each of these factors into consideration, researchers have developed numerous methods to enhance drug absorption across the skin. Figure 6 presents a summary of the strategies employed for the enhancement of transdermal drug delivery systems. In this paper, the enhancement strategies are classified by pairing the type of methods previously reported by Barry [43] and Morrow et al. [73] with their generation, categorised by Prausnitz and Langer [10].

The first generation of transdermal drug delivery does not involve a patch system instead the drug is formulated into a conventional liquid spray, gel, cream or other topical formulations. These formulations are applied on the skin without the involvement of any sophisticated systems or platforms [10]. This method utilises passive diffusion to achieve absorption into the skin and so the incorporated drug must be of low molecular mass (< 600 Da), possessing sufficient hydrophobicity and must be effective in low dose administration [74]. Some marketed transdermal products, as listed in Fig. 5, are examples of this first-generation technology, such as Duragesic^®^ and Salonpas^®^. However, the delivery of drug using this method is very limited, hence some more advanced enhancement methods were subsequently developed. Barry [43] and Morrow et al. [73] have categorised the approaches for enhancing transdermal drug delivery into five methods, as indicated in the purple-shaded semi-circle in Fig. 6.

### Drug-vehicle interaction

The first method which can be used to improve skin absorption is drug-vehicle interaction. Specifically, this method is divided into four different techniques: drug/prodrug selection, ion pairing, eutectic systems and chemical potential or thermodynamic methods. These techniques were all classified by Prausnitz and Langer [10] as second-generation transdermal drug delivery systems. A second-generation strategy was aimed at increasing skin permeability by modifying the SC and providing an additional driving force to across the skin, using methods which avoid damages to the deeper skin layer. In this section, prodrug and ion pairing methods will be utilised as examples of drug/vehicle interactions.

The prodrug approach/technique involves the linking of an inactive moiety to a drug, using covalent interactions, so that the modified drug (parent drug) is more hydrophobic than the active form [73]. This modification is important, since the principal SC barrier is composed of nonpolar lipids. After administration, the parent drug will be metabolised and converted into the active drug [48]. Previous studies have shown that utilisation of the prodrug method can improve the pharmacological activity of certain drugs. Some representative drugs which have been investigated using this approach are stavudine [75], naltrexone [76], bupropion [77], morphine [78], indometacin [79], carbamate [80] and haloperidol [81].

The next method for increasing drug permeation into the skin is ion pairing. This method is suitable for ionised drugs, since they are not readily absorbed via the/across the SC [43]. The addition of the opposite ion species into the drug formulation will result in a neutral paired compound that has a different partition coefficient to the SC [73]. Following application of the ion-paired molecules, the parent drug will be released and then absorbed into the circulation [74]. The release of the drug is caused by the partition and diffusion of the ion-pair through the SC prior to dissociation in the viable epidermis. Some previous studies have reported that the delivery of the following drugs was successfully enhanced using the ion pairing method: risedronate [82], bisoprolol [83], escitalopram [84], berberine [85], zaltoprofen [86] and nicotine [87].

Despite all the advantages documented above, it is worth noting that drug-vehicle interaction methods have some drawbacks. For instance, a prodrug may result in toxicity because of unpredictable metabolism, potentially leading to the production of toxic metabolites [88]. Moreover, it is imperative to assess the toxicity of the linking agent (inactive moiety) and the prodrug itself, due to the possibility of toxic side products upon synthesis [89]. Furthermore, the production of a prodrug requires complex fabrication considerations/methods and is limited only to the generation of small molecules [10]. In addition, the delivery of both ion-pairs and prodrugs are still reliant on passive diffusion techniques and, therefore, the SC still remains a challenge when considering the transdermal delivery of drugs in these forms.

### Vesicles and analogues

**Fig. 7:**
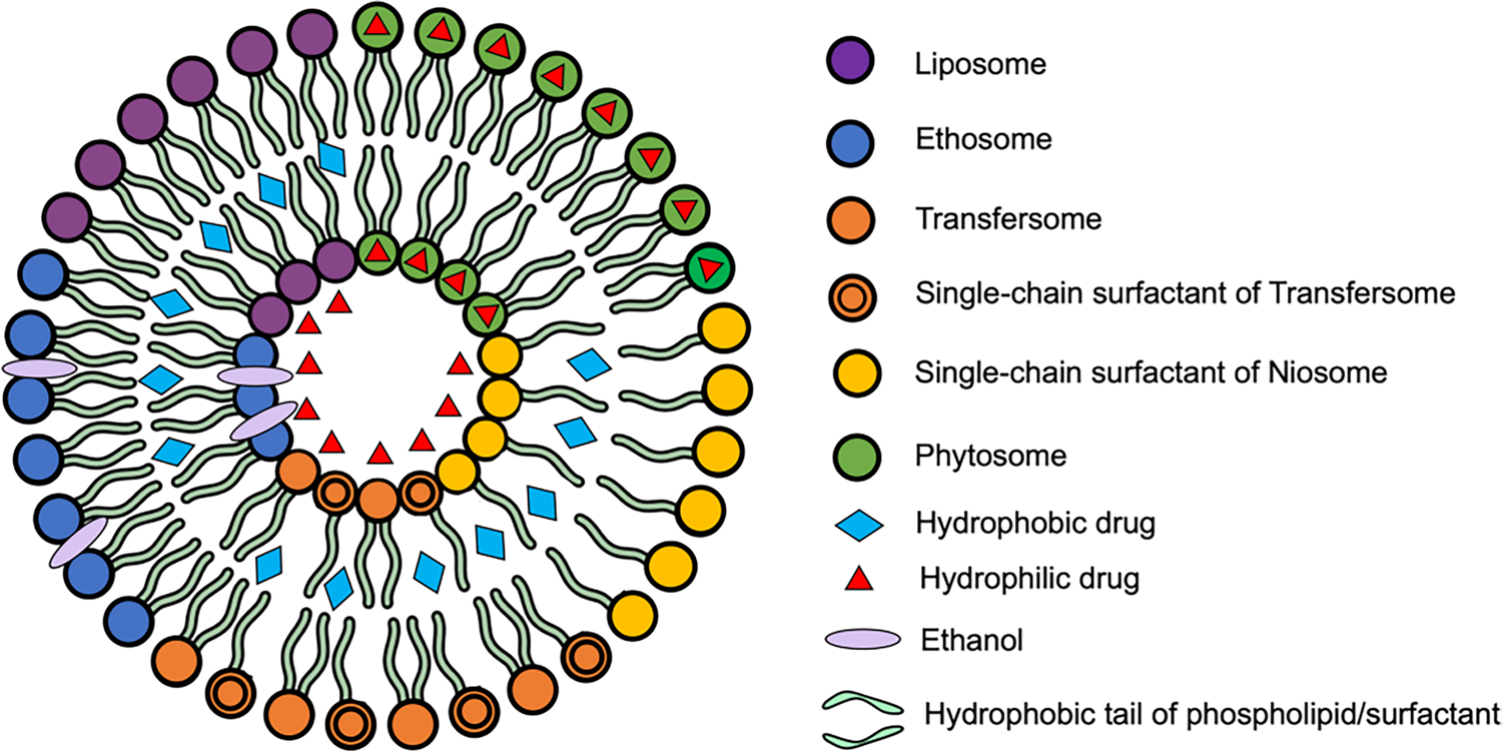
A schematic illustration of a variety of different nanovesicles developed for use in transdermal delivery systems

The second approach for enhancing transdermal drug delivery is the utilisation of vesicles and their analogues. In 2008, this strategy was categorised as the second generation of transdermal products [10]. Nanovesicles are defined as nano-size spherical bilayer vesicles made up of lipids or other analogues, such as surfactant [90]. Some nanovesicles used to facilitate transdermal delivery include liposomes, ethosomes, transfersomes, niosomes and phytosomes. These nanovesicles are classified based upon the main component used in the formulation. Different types of nanovesicles are described and summarised in Fig. 7.

Liposomes were the first artificial vesicles developed by Bangham and Horne in 1964, composed of phospholipid and cholesterol [91]. Liposomes may be composed of one or more bilayer concentric membranes, namely unilamellar vesicles (ULVs) or multilamellar vesicles (MLVs), respectively [92]. Phospholipids, as the main component of liposomes, are amphiphilic molecules, composed of a polar head and a nonpolar tail [93]. Therefore, liposomes can be used for encapsulation of both hydrophilic and hydrophobic drugs [94]. When the drug is hydrophilic, it will be entrapped in the core (interior) of the liposome vesicles, as depicted in Fig. 7. Conversely, a hydrophobic molecule will be encapsulated in the middle of the lipid bilayer that is constructed of nonpolar tails of the phospholipid [95]. Liposomes will be absorbed onto the skin and fuse with the lipid bilayer of SC, which resulting in the disruption of the outer layer integrity. Therefore, the lipids act as skin penetration enhancers that facilitate drug permeation into skin [73]. Liposomes have been used for the delivery of various different kinds of drugs, such as diclofenac [96], baicalein [97], amphotericin B [98], ketoprofen [99], vitamin C [100] and azithromycin [101].

The second type of nanovesicle utilised for transdermal drug delivery systems is the ethosomes. Ethosomes were first developed by Touitou et al*.* [102]. The main composition of ethosomes is phospholipid and alcohol (20–45%), such as ethanol or isopropyl alcohol [102]. The use of ethanol in ethosomes was aimed at improving the flexibility of conventional liposomes and functions as an enhancer upon permeation of the drug into the skin [103, 104]. Ethosomes were deemed to possess better biocompatibility and higher drug permeabilities, compared to conventional liposomes [105]. Some representative drugs which have been successfully delivered using ethosomes include the following: apigenin [106], valsartan [107], econazole nitrate [108], quercetin [109], indometacin [110], curcumin [111], green tea extract [112] and mitoxantrone [113]. A modification of ethosomes, namely transethosome, was introduced by Song et al*.* [114] who combined the use of phospholipids, with high amounts of ethanol and surfactant, acting as permeation enhancers, for delivery of voriconazole. This technology has been investigated for the delivery of fisetin [115], piroxicam [116], paeonol [117], epigallocatechin gallate-containing extract [118] and agomelatine [119].

Transfersomes are ultra-deformable liposomes that were invented by Cevc and Blume and were composed of phospholipid and edge activator (single chain surfactant) [120]. The edge activator is a component that destabilises the lipid bilayer and makes transfersomes much more flexible and deformable, compared to a conventional liposomes [121]. This deformable structure allows the transfersomes to penetrate into the deeper skin layers, using elastic transport [122]. Additionally, skin hydration and osmotic mechanisms are also involved in the penetration processes of transfersomes [123]. Similar to liposomes, transfersomes can encapsulate both hydrophobic and hydrophilic drugs (Fig. 7). Recent studies have shown that cilnidipine [124], diflunisal [125], sinomenine [126], green tea extract [127], pentoxifylline [128], raloxifene [129] and minoxidil [130] were all successfully formulated and delivered transdermally using transfersomes.

Surfactant-based nanovesicles called niosomes can also be used for improving transdermal delivery. Niosomes are mostly prepared with single chain nonionic surfactants using a hydration method to form a bilayer structure [131]. In terms of composition, niosomes can also contain cholesterol to form a rigid structure [132]. Two kinds of nonionic surfactants which have been employed in the manufacturing of niosomes are Tween and Span [133]. Similar to phospholipid, nonionic surfactants also consist of hydrophilic heads and hydrophobic alkyl chains (tails). Hence, niosomes can be used for incorporating both polar and nonpolar compounds [134]. Niosome systems have been reported to enhance the transdermal delivery of salidroside [135], sulfadiazine [136], capsaicin [137], resveratrol [138], atenolol [139] and sumatriptan [140].

The final type of nanovesicles developed for enhancing drug absorption via the skin is the phytosomes which are lipid-based nanovesicles designed to facilitate delivery of hydrophilic phytoconstituents [141]. Phytosomes are also known as phyto-phospholipid complexes due to the complexation of active constituents from plant extracts and phospholipids by covalent interactions [142]. Thus, the entrapment of drug molecules in phytosomes is different from liposomes and other analogues, such as ethosomes or transfersomes [143]. In phytosomes, the hydrophilic drug is entrapped in the polar head of the phospholipid as a complex, while in liposomes the drug is encapsulated in the interior of the vesicles, as presented in Fig. 7. Phytosomes have been utilised for increasing bioavailability of natural compounds, such as curcumin [144], sinigrin [145], *Moringa oleifera* extract [146], *Centella asiatica* [147] and 18ß-glycyrrhetinic acid [148].

Although there are numerous prospective uses of nanovesicles in facilitated transdermal drug delivery, there are also some limitations associated with this technology, such as instability of manufactured products, batch reproducibility, large-scale production, low drug loading and maintaining the particle size during preparation [149]. Furthermore, high cost instrumentation and methods are required for manufacturing of these nanovesicle-based technologies [90]. Moreover, it has been previously reported that, liposomal technology can hinder the penetration of small molecules through the skin [150]. Therefore, other novel enhancement strategies have also been developed to facilitate transdermal delivery of drugs.

### *Stratum corneum* modification

Modification of the properties of the SC may lead to improved permeability of drugs into the skin. Morrow et al*.* [73] categorised skin hydration and the use of chemical enhancers as methods for modifying the SC. Based on this classification, these methods are included in the second generation of transdermal delivery (Fig. 6), or in other words, modifying SC without causing any skin damage [10]. Skin hydration is a process to increase skin humidity and water content, so that the drug can more easily permeate into the SC. Several methods which have been employed for maintaining water content, such as the use of occlusive dressing and patches; preventing water loss by adding lipid excipients to the formulation and increasing skin humidity using humectants [43]. Tan et al*.* [151] investigated the effects of occlusive wet hydration patches on the structure of the SC. They found that, after 6 h of hydration, separation of the lipid bilayer of the SC occurred, thus altering the permeability of porcine skin significantly. Moreover, Paudel et al*.* [152] has previously reported that skin hydration, using an occlusive system, may cause a reduction in diffusional resistance of the skin to xenobiotics. In addition, the use of such systems may also positively affect the flux of numerous drugs by increasing the amount permeated and ultimately affecting the absorption rate into the skin [153].

**Fig. 8:**
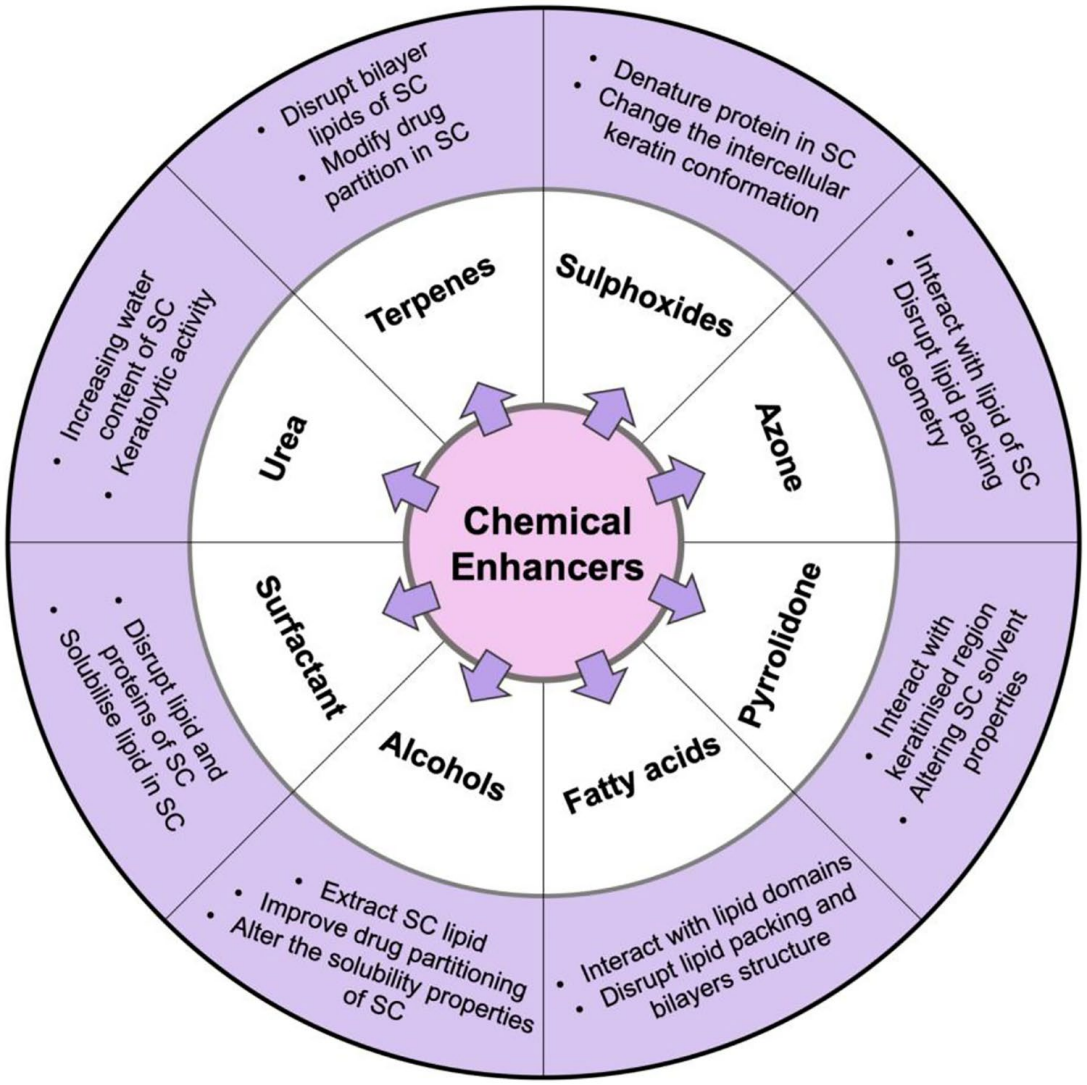
Classification and mechanisms of action of a variety of different chemical enhancer groups used in the facilitated delivery of drugs transdermally (SC: *Stratum corneum*)

Modification of the SC can also be achieved using chemical permeation enhancers. Chemical enhancers function by disrupting the lipid bilayer of the SC, interacting with proteins or modifying the partition coefficient of the drug [74]. These compounds have been widely used in many transdermal products to increase drug permeation. It is necessary, however, to consider the safety of such chemicals. Thus, not all types of chemicals can be used as transdermal enhancers as there are specific requirements for their use in pharmaceutical products. These chemicals must be inert, nontoxic, nonallergenic, nonirritant, preferably elicit rapid effects, be aesthetically accepted and crucially skin barrier function must recover quickly after the chemicals have been removed [154, 155]. Figure 8 summarises the information available from published sources, highlighting the chemical enhancer groups used, to date, in transdermal drug delivery systems [10, 43, 73, 74, 155].

Looking at one example from the sulphoxides, dimethyl sulphoxide (DMSO) has been used to enhance drug absorption by interacting with lipid domains of the SC [73]. DMSO may also denature the protein components and change the intercellular keratin conformation of the SC [155]. Recently, DMSO has been investigated for enhancing transdermal delivery of hydrophilic drugs by altering the diffusivity in the SC corneocytes [156]. Several drugs, including fenoterol hydrobromide [157], hydrocortisone [158], testosterone [159] and naloxone [160] have been successfully delivered transdermally using DMSO as a permeation enhancer.

A second group of chemical enhancers known as azone (1-dodecylazacycloheptan-2-one) can be used as a permeation enhancer, interacting with lipids of the SC and disrupting the lipid packing arrangement of the bilayer [155]. Azone has been utilised for delivery of levamisole hydrochloride [161], ketoprofen [162], dimethyl fumarate [163] and 5-fluorouracil [164]. Even though azone is effective at low concentration as an enhancer, this compound has been still investigated its metabolism in the body and, therefore, has not ever been used in commercial products [155]. Other synthetic chemical permeation enhancers are from the pyrrolidone group, including n-methyl pyrrolidone and 2-pyrrolidone. These chemicals can interact with the keratinised region of the SC and alter the solubility properties of the SC [73]. N-methyl pyrrolidone has been investigated for delivery of ketoprofen [162], lidocaine hydrochloride [165], bupranolol [166] and 5-hydroxymethyl tolterodine [167] via transdermal route.

Fatty acids can also act as chemical permeation enhancers. As previously explained, the SC is composed of lipids and possesses hydrophobic characteristics. Therefore, the usage of fatty acids, such as oleic acid and lauric acid, will increase drug permeation through the skin due to the similar hydrophobicity, compared to the lipids of the SC. These fatty acids will interact and modify lipid domains of the SC by disrupting lipid bilayer packing [153]. Flurbiprofen [168], propranolol [169], theophylline [170] and donepezil [171] are examples of drugs, the transdermal delivery of which was enhanced using fatty acids.

Alcohols, such as ethanol, propylene glycol and isopropyl alcohol, also act as skin permeation enhancers. These solvents can increase drug solubility in the SC by altering the solvent properties of the SC, resulting in improvement of drug partitioning [43]. Moreover, a gradient concentration mechanism is also involved in this enhancement, as alcohols evaporate quickly after application [155]. Additionally, ethanol may also cause slight disruption of the intercellular lipid geometry of the SC [73]. Alcohol has been used for improving transdermal permeation of several drugs, such as thyrotropin releasing hormone [172], nortriptyline hydrochloride [173], thymoquinone [174] and lidocaine [175].

Surfactants are amphiphilic molecules which have both polar and nonpolar functional groups [155]. Surfactants can solubilise the lipids of the SC, disrupting the lipid and protein domains and penetrate through the lipid bilayer [176]. Surfactants which have been widely used for enhancing transdermal absorption of drugs are sodium lauryl sulfate (SLS) and polysorbate (Tween). SLS was studied as a means of increasing transdermal delivery of lorazepam [177] and foscarnet [178], while polysorbate has been reported as an enhancer of the transdermal delivery of L-ascorbic acid [179] and dimethyl fumarate [163]. Similar to the surfactant group, urea is also employed for improving skin absorption of drugs by disrupting SC lipids, increasing water content of the skin and initiating keratolytic activity [153]. Urea has been investigated as an enhancer of transdermal drug delivery in some formulations containing indometacin [180], venlafaxine hydrochloride [181] and metronidazole [182].

The last group of chemical enhancers that will be explained in this section is the terpenes group. Terpenes are volatile compounds (mostly extracted from natural products) which are constructed of carbon, hydrogen and oxygen atoms [183]. Monoterpenes (C_10_) and sesquiterpenes (C_15_) are two kinds of terpenes with high percutaneous enhancement activities. Limonene, cineol, menthol and carvacrol are included to the monoterpenes class, while bisabolol, farnesol and nerolidol are in the sesquiterpenes class [183]. The mechanism of action of terpenes as permeation enhancers is by modifying the solvent nature of the SC and improving drug partitioning into the SC [155]. Moreover, terpenes can also disrupt the SC lipid bilayers and modify diffusivity of drug delivered [73]. Chen et al*. *[183] have summarised additional modes of action of terpenes, such as extracting part of the SC lipids, denaturation of keratin and disordering the lipid arrangement in the SC. Terpenes have been investigated in in vitro studies as permeation enhancers for transdermal delivery of propranolol [184], pizotifen [185], zidovudine [186], dimethyl fumarate [163] and imipramine hydrochloride [187].

In spite of these enhancement methods showing significant promise, there are some disadvantages associated with the use of chemical enhancers. For example, some of the chemicals are toxic and may cause skin irritation when used at high concentrations, such as DMSO [155]. Moreover, chemical enhancement is a concentration-dependent method. Hence, it may prove ineffective at particular concentrations [73]. Additionally, the effective concentration for each type of chemical enhancer is different for each drug [188]. As such, a combination approach, using different types of chemical enhancers has been employed to investigate the different ways of increasing drug permeation across the skin (Fig. 6). This combination has been categorised as the third generation of transdermal enhancement methods [10].

### Energy-driven methods

Energy-driven or electrically assisted methods of transdermal delivery use electrical devices for enhancing absorption of drugs into the skin. Based on Prausnitz and Langer’s classification [10] and Morrow et al*.* [73], the energy-driven methods are divided into two generations, as previously displayed in Fig. 6. The second generation of electrically assisted methods are iontophoresis and noncavitational ultrasound, whereas the third generation are electroporation, sonophoresis and thermal ablation. Prausnitz and Langer [10] classified the iontophoresis as the second generation, since this technology does not give a large impact on the SC, such as microchannel creation or SC removal, as found in electroporation and microscissioning, respectively. In contrast, the third generation of enhancement technology requires stronger disruption of the SC, because the main target of these methods is to disrupt or remove the SC, without causing damage to the deeper skin tissue [10]. In this review article, the second-generation energy-driven methodology, iontophoresis, and the third-generation methods of sonophoresis and electroporation will be described as examples of these approaches.

**Fig. 9:**
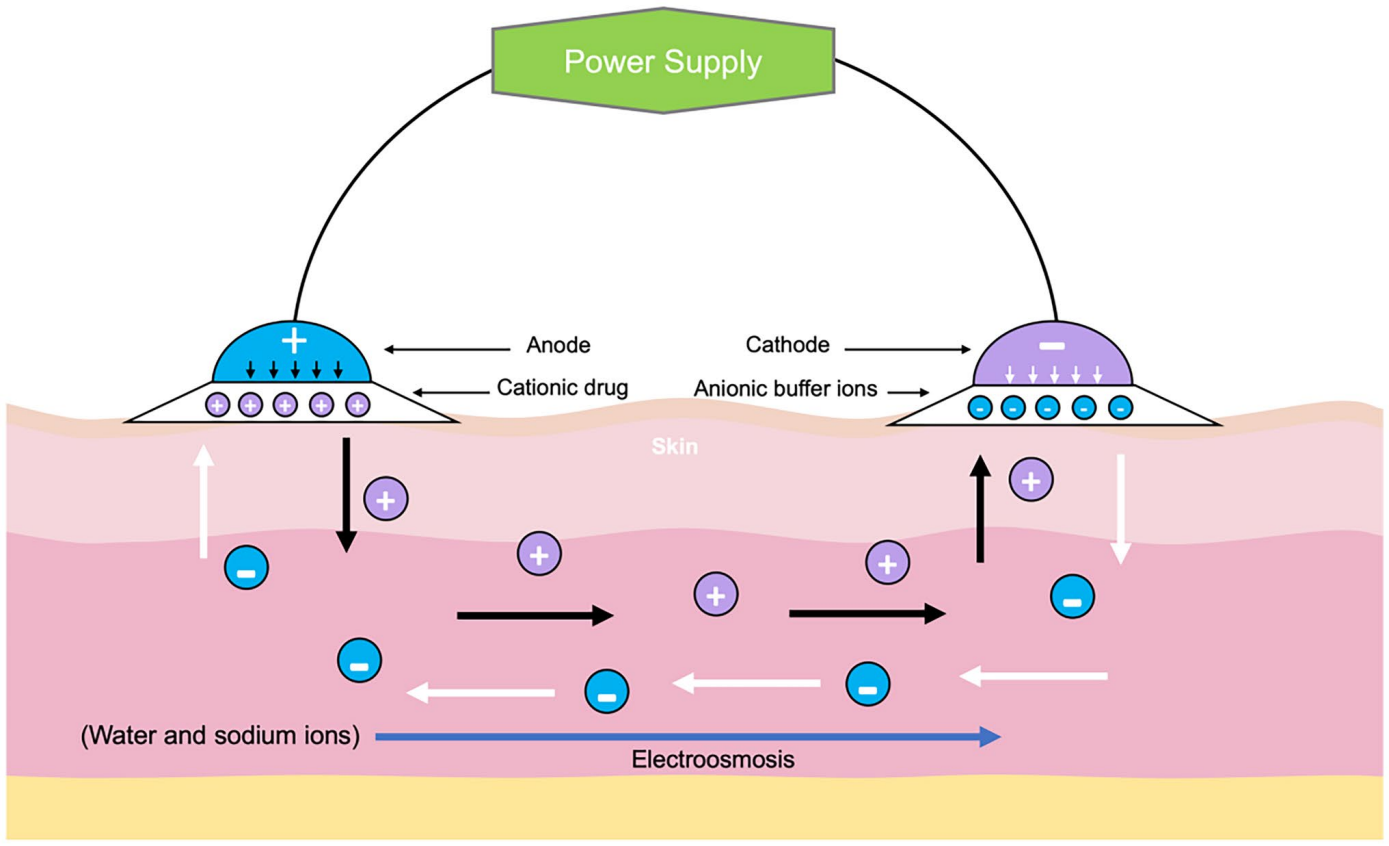
Schematic diagram illustrating the delivery mechanism of cationic drugs using iontophoresis. The black arrows in the skin describe the flow of cationic drug moves from drug solution to the cathode. Conversely, the white arrows show the movement of anions from buffer solution under the cathode to the anode

Iontophoresis is defined as a method for increasing permeation of drugs into the skin using small electrical currents [189]. The electrical current applied in iontophoresis varies from 0.5 to 20 mA [190]. The principle of this system is based on the different charges of the electrodes used, namely the anode and cathode. Anionic drugs are placed underneath a cathode, and the cationic or neutral drugs are placed ions under the anode. Upon application of low electricity current at low voltage, ions permeate into the skin [73]. In the skin, the anions are mobilised to the anode by a repel pulse from the cathode, and vice versa for the cation, as illustrated in Fig. 9. Iontophoresis is mostly used for increasing skin absorption of ionisable drugs [191]. However, this technology can also be used for delivery of weak charged or neutral molecules [10] through electroosmosis [73]. There are some factors that may affect drug delivery via iontophoresis, such as formulation, physiology of the application site, physicochemical characteristics of the drug delivered, duration of application and the instrument parameters [191]. Iontophoresis has been investigated for delivery of various kinds of drugs, including nonsteroidal anti-inflammatory drugs (ibuprofen, aspirin and indometacin) [192], almotriptan [193], granisetron [194], sodium nonivamide acetate [195], donepezil [196] and insulin [197]. Despite the wide utilisation of iontophoresis, this system has some associated limitations, such as the possibility of skin irritation and current-induced damage to the skin, the requirements of instrument setup, their complexity of use for patients, the drug ions may not easily permeate into the skin and the drug delivered may be limited to the small molecular weight (MW) drugs (< 10,000 Da) [198]. Moreover, the electrodes tend to corrode upon storage due to the aqueous gel nature of the adhesive [199]. The absence of any commercial iontophoresis devices on the market for drug delivery may be caused by the difficulty to predict the complex bioavailability of drugs delivered from iontophoretic product [199]. Furthermore, this delivery platform is more expensive compared to the other types of marketed transdermal products [199].

**Fig. 10:**
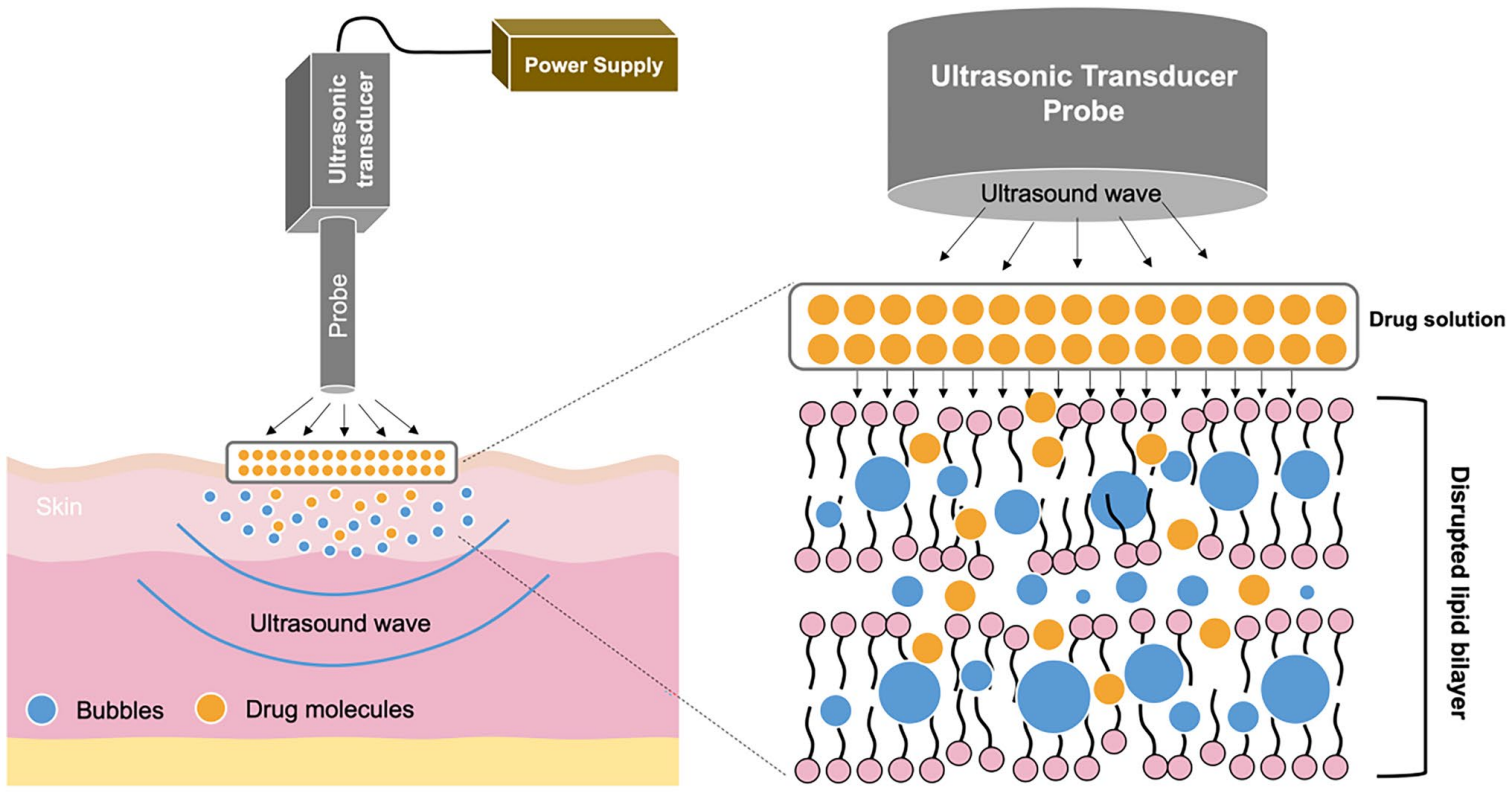
A schematic illustration of sonophoresis-assisted transdermal drug delivery. Following the application of ultrasound, the drug molecules will be delivered into dermis

The next method for enhancing transdermal absorption is sonophoresis, also known as phonophoresis. This system uses ultrasound at frequencies of 20 kHz–16 MHz for modifying lipid bilayer arrangement of the SC [200]. There are two possible enhancement mechanisms of action via sonophoresis, namely, thermal effects and cavitation (including stable and inertial cavitation) [201]. By applying ultrasound, the skin temperature increases, resulting in higher drug diffusivity into the skin [202]. The cavitation mechanism refers to the formation of cavities and bubbles in the SC. When ultrasound is applied, it causes a continuous oscillation and a stable cavitation, inducing bubbles around the application area [201]. This mechanism is termed stable cavitation. Inertial cavitation is defined as the creation of bubbles inside the liquid medium in a single or multiple cycles upon the application of ultrasound [201]. In sonophoresis, the drug solution is placed under the probe equipment and then a predetermined ultrasound frequency is applied onto the drug solution and skin [203]. Upon ultrasound exposure, the cavities and bubbles are created, causing the disruption of lipid bilayer packing in the SC (particularly in the interface of keratinocytes and lipids of SC) [204]. Figure 10 depicts the mechanism of drug release using sonophoresis.

Drug delivery systems using sonophoresis are influenced by a number of factors, such as the frequency, intensity and mode of ultrasound [205]. Several other parameters that may affect the success of sonophoresis are application time of the ultrasound, coupling medium, and distance of ultrasonic probe from the skin [206, 207]. Sonophoresis is beneficial for the delivery of a variety of different kinds of drugs. For instance, it has been reported that sonophoresis was successfully employed as a transdermal enhancement method in the delivery of ketoprofen [207], fluocinolone acetonide [208], vancomycin [209], gemcitabine hydrochloride [210] and proteins (insulin, interferon y, and erythropoietin) [211]. Many different classes of drugs have been successfully delivered using this approach. This indicates that sonophoresis can be used to facilitate the delivery of hydrophobic and hydrophilic drugs but also small molecules and macromolecules. There are some disadvantages associated with sonophoresis, however, such as the time-consuming nature of the technique, requirement for sophisticated instrumentation and skin must be in a healthy condition during application [212].

**Fig. 11:**
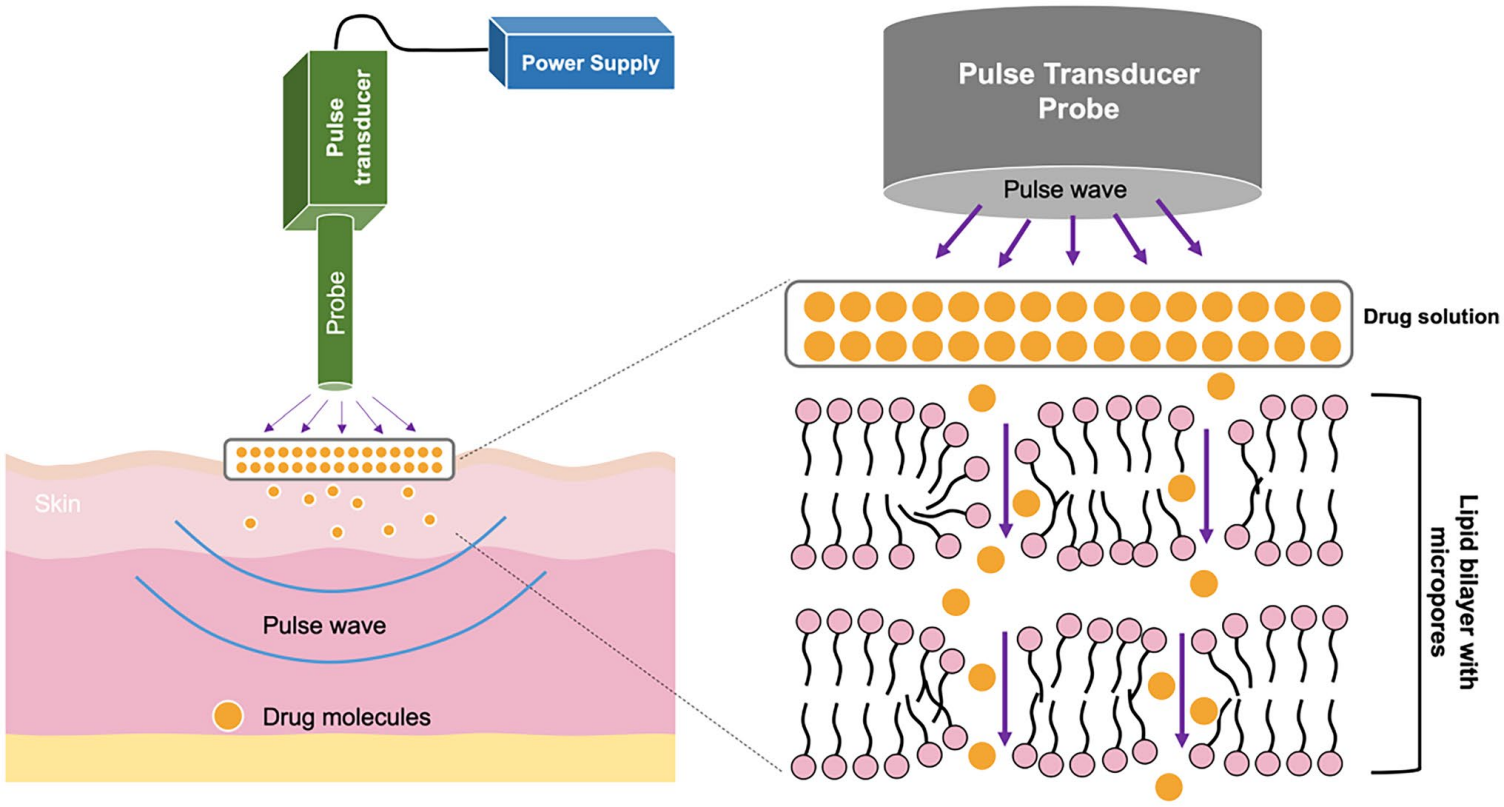
Schematic diagram illustrating transdermal drug delivery using the energy-driven electroporation method. Electroporation helps the drug to permeate into the dermis layer

The final example of an energy-assisted enhancement method is electroporation. Electroporation is a technique utilised to create micropores in the skin by applying high voltage (10–1000 V) over a very short time period (less than a few hundred milliseconds) [73]. The principle of electroporation involves exposing a drug solution, which has been placed on the skin, to pulse waves [213]. This pulse wave will create aqueous pores in the lipid bilayer of the SC and allow drug penetration into the deeper skin layers via the pores created, as detailed in Fig. 11. Therefore, this method holds the potential to deliver macromolecules across the skin as the pores are created in the SC itself.

Drug delivery using electroporation is affected by several factors, for example the physicochemical properties of the drug, voltage used, length of pulse and number of pulses [213]. Electroporation has been employed to enhance the in vitro transdermal absorption of various drugs, including tetracaine [214], alniditan [215], insulin [216], fentanyl [217], timolol [218] and calcein [219]. An example of transdermal in vivo delivery using electroporation was reported by Blagus et al. [220] who investigated the permeation of dextran, doxorubicin and fentanyl in a rat model. Nevertheless, electroporation may suffer from some limitations, such as the need for sophisticated and high-cost instrumentation, complicated and time consuming means of application, the necessity for expert personnel, low throughput of the drug delivered due to the limited area of aqueous pores created, the possibility of cell damage because of high voltages utilised [212, 221]

### *Stratum corneum* bypassed

Innovative enhancement technologies aimed at bypassing the SC have also undergone considerable research and development. In this section, the use of bypass methods will be explored, facilitating transdermal delivery of a wide variety of drugs. A skin bypassed technique which is included to the second generation of enhancement technology is transfollicular delivery (Fig. 6). Transfollicular delivery refers to drug delivery via hair follicles as the principal absorption route [222]. As previously explained, however, this route is limited by the area of hair follicles on the skin, which is only ~ 0.1% of the skin surface area [43].

**Fig. 12:**
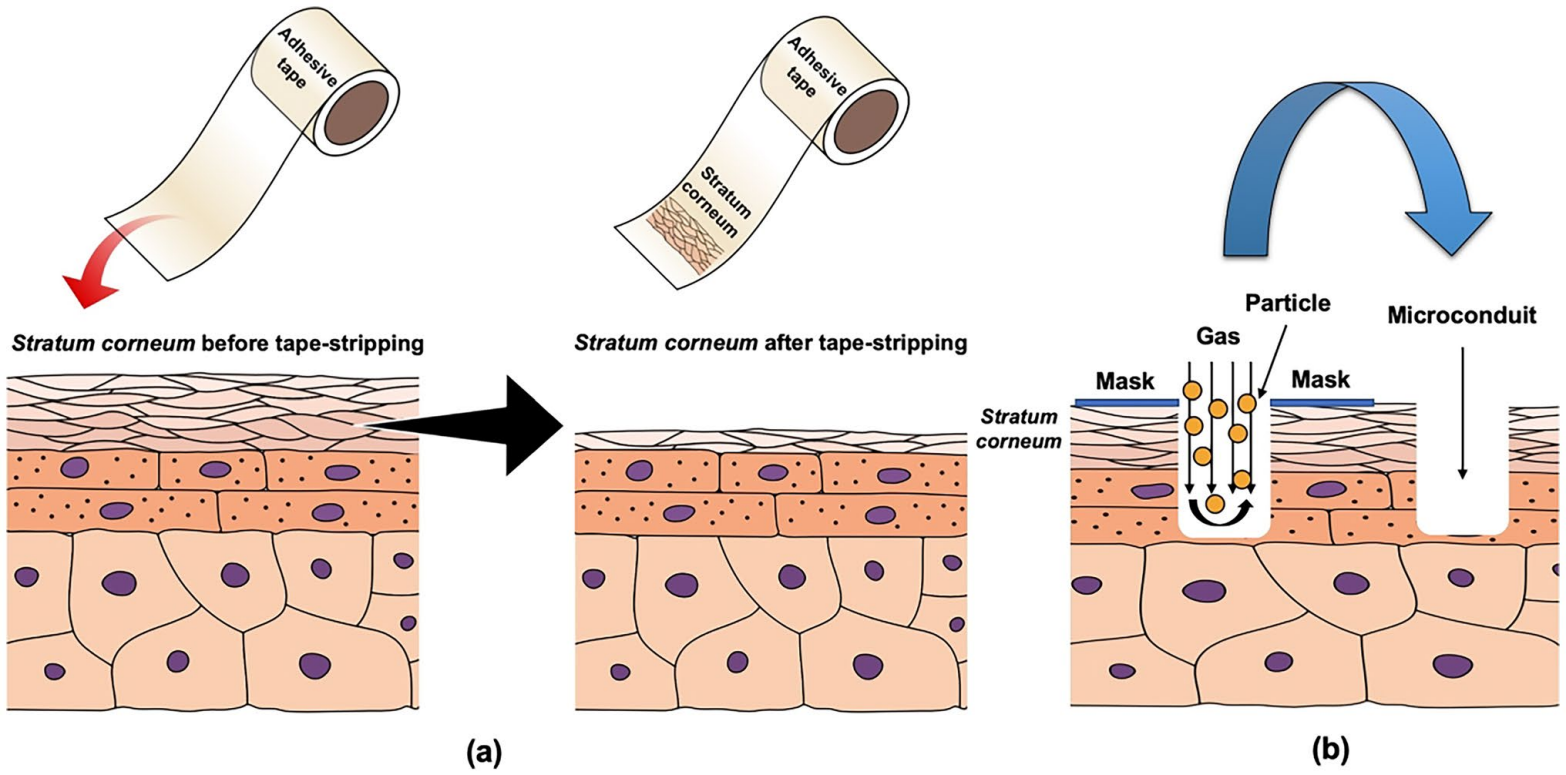
Diagram illustrating the process of (**a**) skin tape-stripping and (**b**) microscissioning

Tape-stripping, microscissioning and microneedles are examples of third generation skin bypassing methods. Tape-stripping is a technique to remove the superficial SC layers by applying adhesive tape on the skin surface for several times [73]. By removing the successive layers of the SC (Fig. 12a), drug permeation into skin may be improved. Despite the simple and cheap method used, such system has a low reproducibility and is inconvenient for regular applications [73]. A more advanced technique for disrupting SC of the skin is by using microscissioning, which was firstly reported by Herndon et al. [223]. In this technique, the SC is removed gradually using aluminium oxide particles which are mobilised in an accelerated velocity. The accelerated particles will cut the SC and create microconduits in the skin (Fig. 12b). Nevertheless, this technique shows some drawbacks, such as time-consuming preparation, particles deposition in the skin and the possibility of infections following the application [73]. The last SC bypassed method employed for improving transdermal drug delivery is microneedles.

## Microneedles

Microneedles (MNs) are micron-sized needles, on a solid support, with needle heights ranging between 25 and 2000 µm. These needles can pierce the SC and create microconduits, following insertion into the skin [49, 224]. The first concept of MN was introduced by Gerstel and Place [225] in their patent, entitled ‘Drug Delivery Device’. This outlined the basic design of MN that continues to undergo development today. This finding was then followed by Gross and Kelly [226] who published a patent about the first hollow needle connected to an expansible-contractible drug chamber for intradermal drug delivery system. In 1997, another discovery was reported by Jang, who proposed a skin perforating device which was aimed at transdermal delivery [227]. Henry et al*.* [228] manufactured solid MN (made of silicon) for transdermal delivery of calcein as a scientific demonstration of MN.

**Fig. 13:**
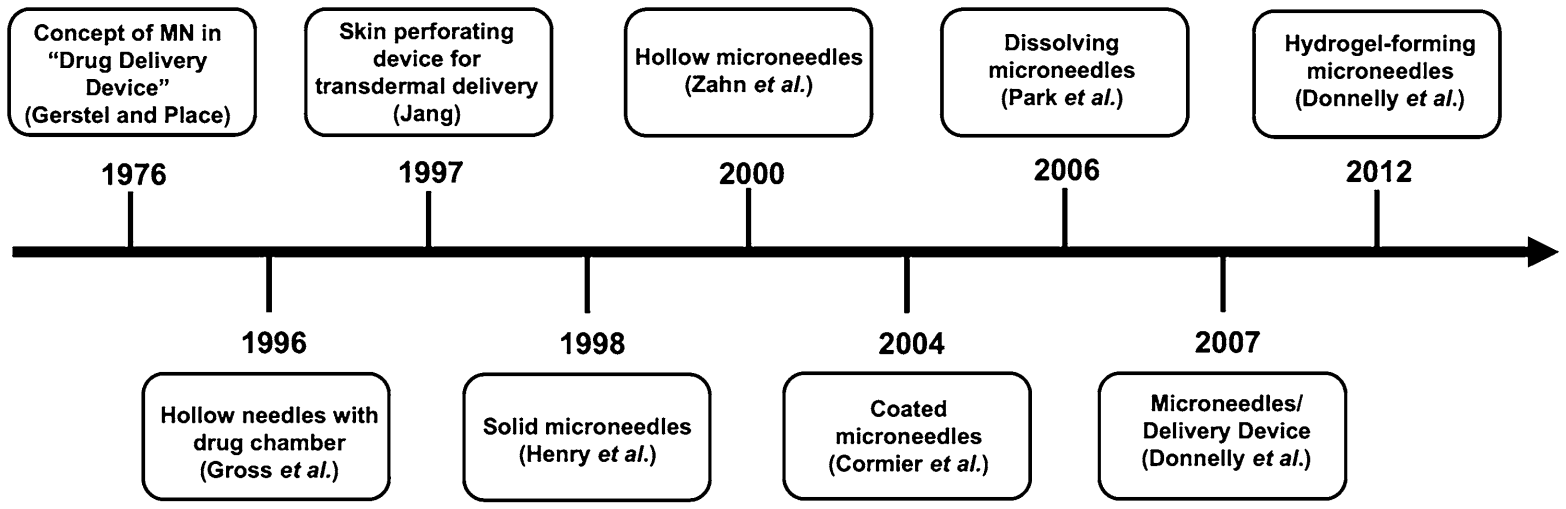
A timeline summarising fundamental findings of MNs since the first conceptualisation in 1976 until the invention of the last MN type in 2012

**Fig. 14:**
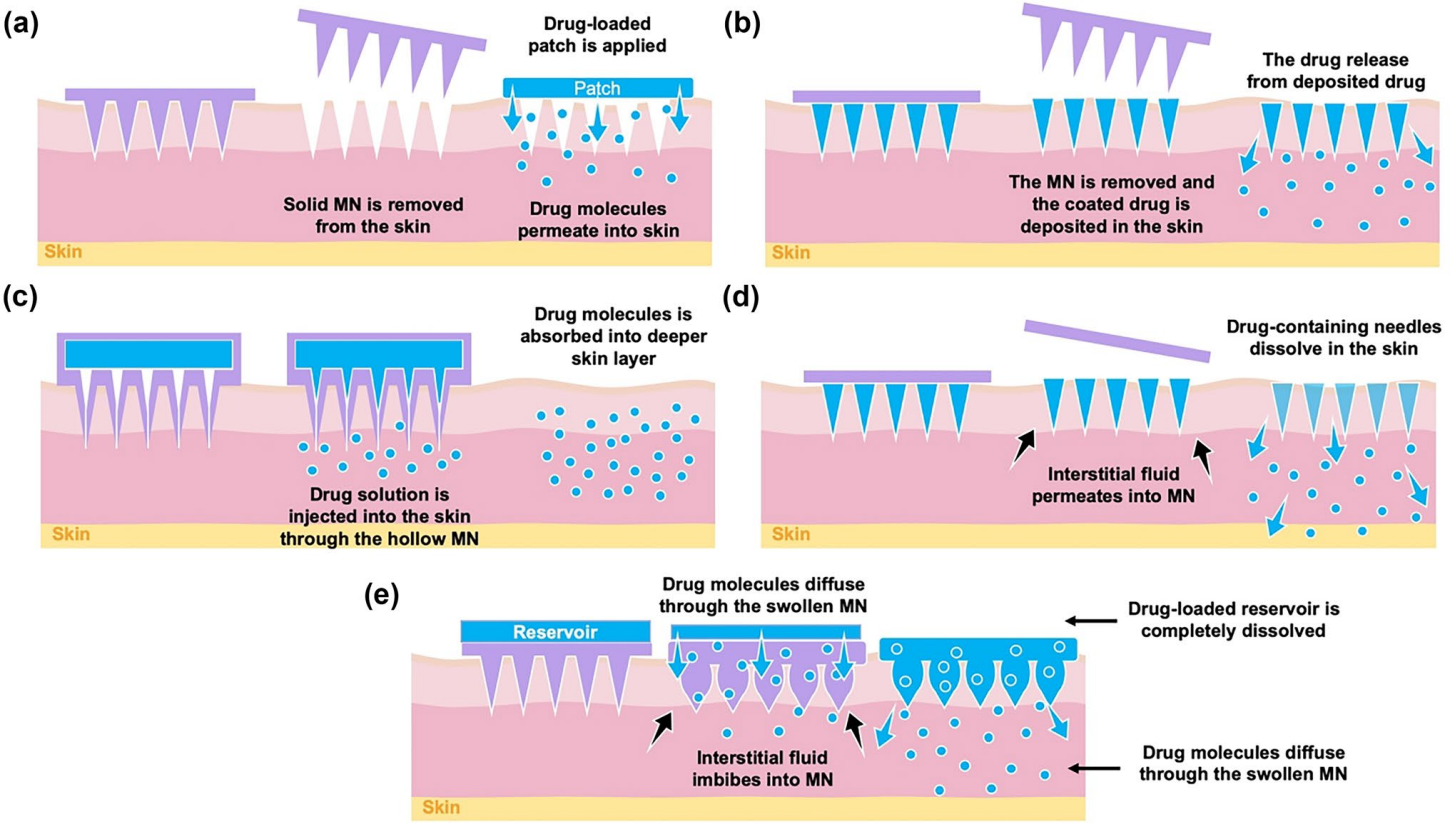
Drug delivery approach via different type of MN: (**a**) solid, (**b**) coated, (**c**) hollow, (**d**) dissolving and (**e**) hydrogel-forming MNs

Following this invention, other types of MNs were developed. Zahn et al*.* [229] invented hollow MN and this technology utilises the ability of MN to penetrate the skin, following which a drug solution is injected through the hollow needles into the skin [224]. Coated MNs were developed by Cormier et al*.* [230] for delivery of desmopressin. In this work, the needle tips of the MN were coated with desmopressin solution and delivery was evaluated in an animal model. A further exploration of MN was investigated by Park et al*.* in 2006. They mixed drug (calcein or bovine serum albumin) with PLGA as the MN material [231]. The drug-containing needles of the MN dissolved upon insertion into the skin, hence this platform was named, dissolving MN. The most recent type of MN was invented by Donnelly et al. [232], namely hydrogel-forming MN. Even though the paper was published in 2012, the patent was filed in September 2007 [233]. This MN is made of hydrogel-forming polymer that can absorb interstitial fluid and swell in the skin after insertion [232]. This technology is completely different from dissolving MN as the drug is not mixed with the MN polymer. A drug-containing reservoir is instead integrated with the MN prior to application. Figure 13 depicts a brief timeline of MN evolution over time, and Fig. 14 is schematic representations of the drug delivery approaches of each different MN.

MNs have been widely used for enhancing transdermal delivery of numerous kinds of drugs. MNs are able to penetrate the SC of the skin and reach the dermis without breaching nerve endings and blood vessels in the dermal layer [49]. Consequently, upon administration, MNs are less painful and more comfortable compared to hypodermic injection. Furthermore, dissolving and hydrogel-forming MNs can avoid the need for sharps waste disposal and does not contribute to transmission of blood borne diseases the way traditional needle and syringe devices can [234]. Moreover, this technology offers considerable benefits over other transdermal enhancement methods explained in the previous section. For instance, since the SC is by-passed, delivery of macromolecules is more feasible using MN. In addition, the skin can quickly recover after the MN is removed [235]; thus, this may prevent irritation or secondary infections at the application site [236]. In terms of materials, MNs are generally manufactured using biocompatible substances or biodegradable polymers [224]. It is important to ensure that MN materials are safe and do not induce inflammation response after the insertion [237]. For routine use, such as in insulin therapy, MNs are possible for self-administration and easy to apply on the skin by patients [238]. The benefits documented above have shown that MNs are designated as a promising strategy to improve drug permeation into the skin. To have a better understanding of MN delivery, each type of MN will be explained in the subsequent sections.

### Solid microneedles

**Fig. 15:**
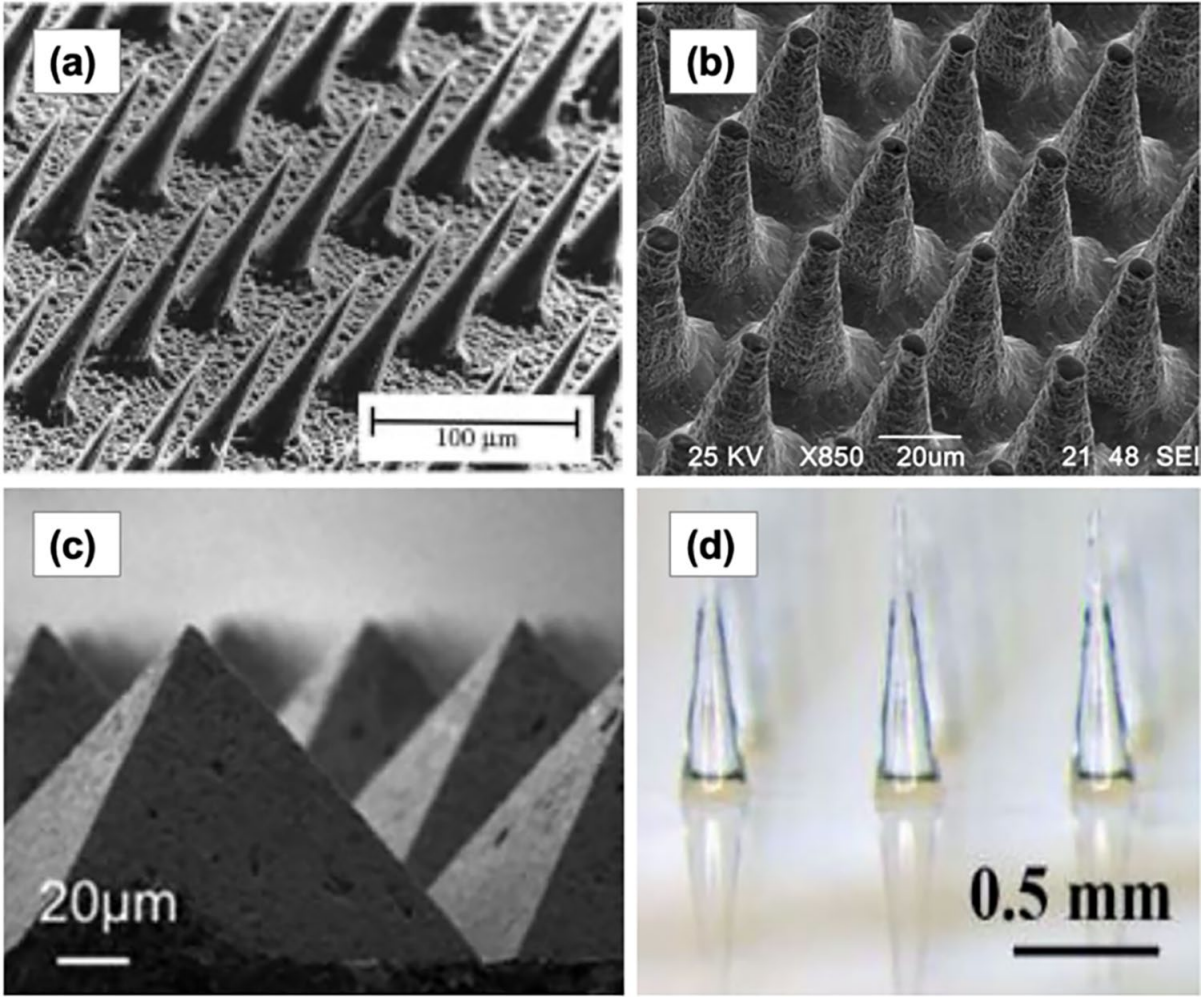
Representative images of solid MNs which were made using (**a**) silicon (reproduced with permission from [228] Copyright 1998, Elsevier), (**b**) tungsten (reprinted with permission from [239] Copyright 2016, American Vacuum Society), (**c**) calcium sulfate dihydrate (reprinted with permission from [243] Copyright © 2014, Royal Society of Chemistry) and (**d**) polylactic acid (reprinted with permission from [244] Copyright © 2017, Royal Society of Chemistry)

The first MN developed for enhancing transdermal drug delivery was the solid MN. Solid MNs use the microconduits created in the skin by the MN as drug absorption channels [224]. Following insertion, the MNs are removed and a transdermal patch is applied onto the microconduits (Fig. 14a). Then, the drugs are released via passive diffusion from the drug formulation and permeate through the microconduits into the skin [224]. As previously explained, solid MNs were primarily made of silicon for delivery of calcein [228]. After this invention, solid MNs were then manufactured using different types of materials, including metal, ceramic and polymers [49]. For example, a tungsten-based solid MN has been manufactured by Ma et al*.* [239], and Tsuchiya et al. [240] has reported a metal-based solid MN fabricated from titanium. Another metal which has been used for manufacturing solid MN is stainless steel. Verbaan et al*.* [241] have used stainless steel-30G hypodermic needles as MN material by cutting and attaching these needles to a poly(etheretherketone) mould. With respect to ceramic-based MN, Bystrova and Luttge [242] manufactured this type of MN by casting alumina slurry into micromoulds. Additionally, different types of ceramic (calcium sulfate dihydrate) were also employed as materials for manufacture of solid MN, as previously described by Cai et al*.* [243]. A solid polymer MN made of poly(lactic acid) was reported by Li et al. [244] for delivery of insulin in a diabetic model rat. Figure 15 displays some solid MNs fabricated using a variety of different materials: (a) silicon [228], (b) tungsten [239], (c) calcium sulfate dihydrate [243] and (d) polylactic acid [244].

Previous studies have shown that solid MN can be used for delivering a wide variety of drugs. A combination of nanoemulsion and stainless-steel solid MN has recently been investigated for improving transdermal delivery of aceclofenac [245]. Moreover, it was reported by Abiandu et al. [246] that the use of solid MN (rollers) can significantly increase in vitro flux of potassium chloride when compared to the passive diffusion group. With regards to delivery of macromolecules, Martanto et al. [247] have used solid MN for increasing transdermal delivery of insulin. The transdermal delivery of some other drugs has been enhanced using solid MN, including bovine serum albumin (BSA) [248], amantadine hydrochloride [249], levodopa [250] and ovalbumin [251]. Despite the wide variety of drugs that can be delivered using this approach, solid MN may have some practical issues associated with them, due to the requirement for a two-step application process [224]. Moreover, some materials are brittle, nonbiocompatible and may induce inflammatory response in the patient [49].

### Coated microneedles

Coated MNs are fabricated by coating the needle tips of the MN with drug formulation [224], either a drug solution or dispersion layer. To prepare the coating, drugs are mixed with thickening agents and surfactants in order to ensure the drugs adhere onto the MN tips prior to insertion into the skin [252]. In terms of materials, the MN can be prepared from metals or polymer, since the basic technology requires the production of a solid MN [252, 253]. Following insertion of the MN into the skin, the drug coating is deposited and dissolves upon contact with interstitial fluid (Fig. 14b).

**Fig. 16:**
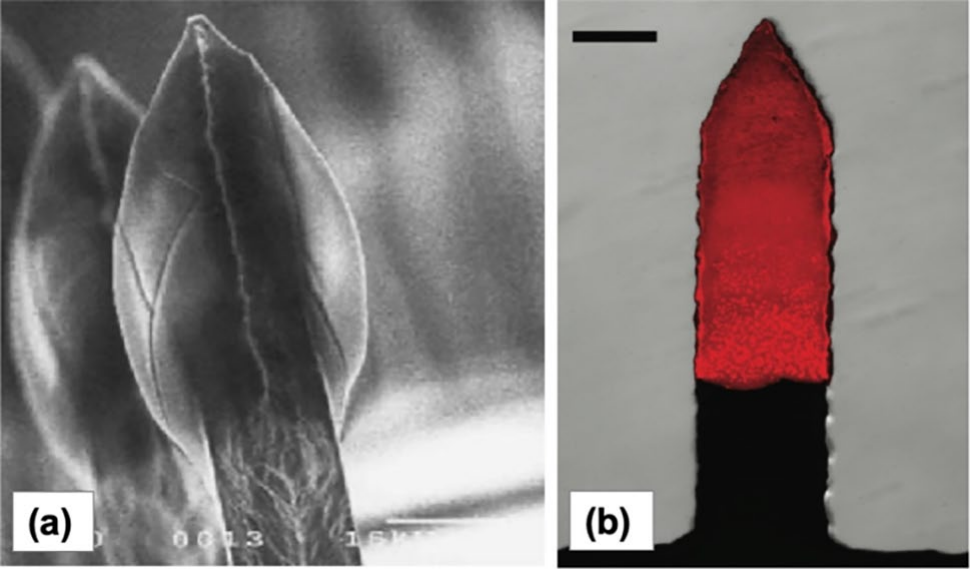
Coated MN with different drug coatings: (**a**) Desmopressin (reproduced with permission from [230] Copyright 2004, Elsevier). (**b**) DNA (reprinted with permission from [255] Copyright © 2010, American Chemical Society)

Coated MNs have been investigated for delivery of macromolecules since their first invention. The first utilisation of coated MN, as previously explained, was reported by Cormier et al*.* [230] for delivery of desmopressin. Subsequently, this type of MN has been used for enhancing the delivery of other macromolecules, such as insulin [254], deoxyribonucleic acid (DNA) [255], exendin-4 [256], botulinum toxin-A [257] and peptide-A [258]. Furthermore, the use of coated MN has also been reported for delivery of lidocaine [259] and riboflavin [260]. The use of coated MN allows a one-step application process which is easier than that required for solid MN. However, the main drawback of using coated MN is the limited amount of drug that can be delivered, due to the constraints/restrictions of the coating surface [224]. Figure 16 shows a series of representatives of coated MN with various kinds of needle shapes and drug coating: (a) desmopressin [230] and (b) DNA [255].

### Hollow microneedles

**Fig. 17:**
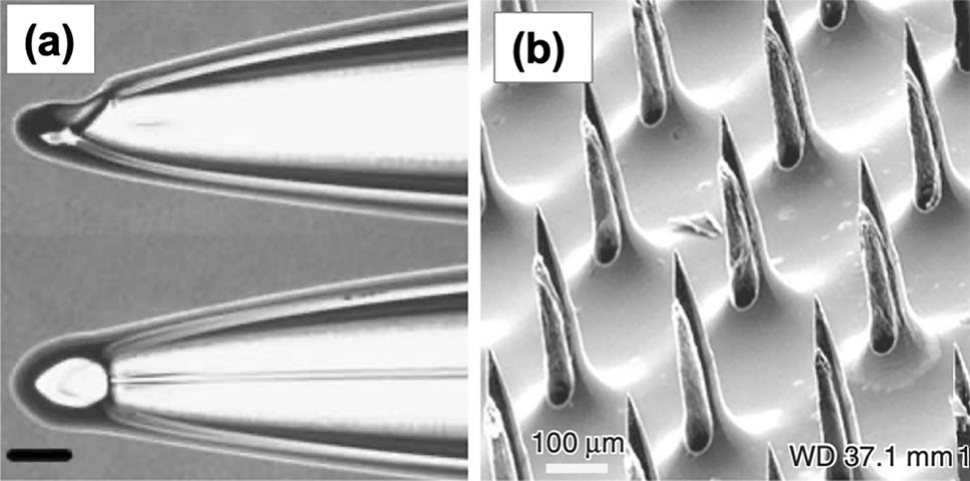
Hollow MNs made of (**a**) glass (reproduced with permission from [267] Copyright 2006, Elsevier) and (**b**) silicon [269] (this is an open access article)

Hollow MNs are designated to deliver drug continuously via needles bores into the skin. In hollow MN, the drug formulation (solution or dispersion) is loaded in the interior of the MN, then the drug is injected and transferred into the skin after insertion of the MN (Fig. 14c). Hollow MNs allow greater amounts of drug loading, when compared to solid and coated MN. Similar to solid MN, hollow MN can be manufactured using metals, silicon, glass or polymers [261]. Shikida et al*. *[262] developed hollow MN using nickel. Silicon- and glass-based hollow MNs have been previously described by Jurčíček et al*.* [263] and McAllister et al*.* [248], respectively. With regard to hollow polymer MN, some materials which have been used for manufacturing this type of MN are poly(oxymethylene) [264], poly(imide) [265] and poly(methyl meta acrylic) [266]. Insulin has been delivered transdermally using hollow MN [267]. However, the use of hollow MN is not limited to transdermal delivery purposes. For instance, Niu et al*.* [268] have investigated intradermal delivery of vaccine nanoparticles using hollow MN in a rat model. The major disadvantage of using hollow MN is the possibility of blockage after the needles are inserted due to the open needle bores. Figure 17 depicts examples of hollow MNs which are fabricated using different materials: (a) glass [267] and (b) silicon [269].

### Dissolving microneedles

Dissolving MNs represent a distinct section of MN technology. Following insertion, the needles dissolve in the skin and the drugs are released gradually from the MN matrix (Fig. 14d). For fabricating dissolving MN, the drugs are mixed with soluble and biocompatible polymers [224]. Thus, drug release rate is mainly influenced by the polymer composition and MN matrices [224]. Dissolving MNs have been manufactured using numerous different polymers, such as poly(lactide-co-glycolide) [231], carboxy methyl cellulose [270], poly(vinyl alcohol) [271], poly(vinyl pyrrolidone) [272], hyaluronic acid [273], pullulan [274] and copolymers of methyl vinyl ether and maleic acid [275].

**Fig. 18:**
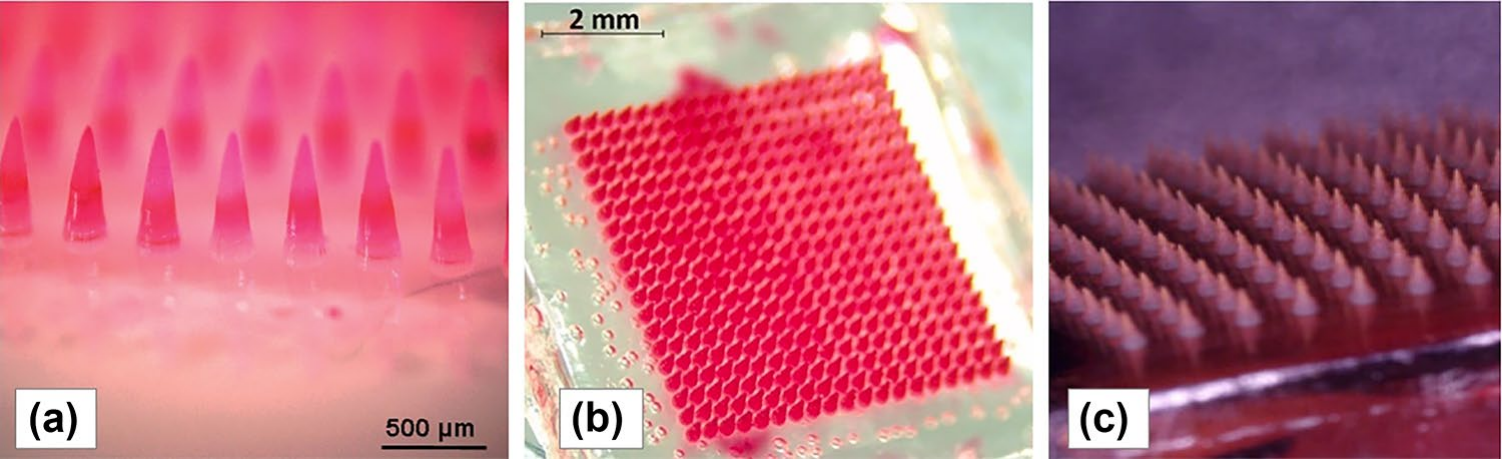
Micrographs of dissolving MN fabricated from (**a**) poly(vinylpyrrolidone) (reproduced with permission from [281] Copyright 2008, John Wiley and Sons), (**b**) poly(vinylpyrrolidone) (reproduced with permission from [277] Copyright 2019, Elsevier) and **c** pullulan [274] (this is an open access article)

Many different drugs have been successfully delivered transdermally using dissolving MN. For instance, this technology has been investigated both in in vitro and in vivo studies for delivery of vancomycin hydrochloride [276], vitamin B12 [277], rilpivirine [271], dutasteride [270], bevacizumab [278], albendazole [279], insulin [261], ovalbumin [273] and sumatriptan [280]. Figure 18 shows some examples of dissolving MN which fabricated from different polymers: (a) poly(vinylpyrrolidone) [281], (b) poly (vinylpyrrolidone) [277] and (c) pullulan [274].

These studies have shown that dissolving MN can be used for delivering both small and large MW drugs. Moreover, the drugs documented above are also varied in terms of polarity. Although dissolving MNs are more suitable for water soluble drugs, this type of MN has also been explored for delivery of hydrophobic drugs in particulate systems, such as solid lipid nanoparticles [279] and microparticles [282]. However, dissolving MN also have some inherent drawbacks, such as the deposition of polymers in the skin, limited amounts of drug that can be formulated in the needles and subsequently delivered into the skin [224]. In a repeat application of dissolving MN, the deposition of high MW synthetic polymers in the skin may induce erythema or hepatic/lymphatic accumulation, as previously described [274, 283].

### Hydrogel-forming microneedles

**Fig. 19:**
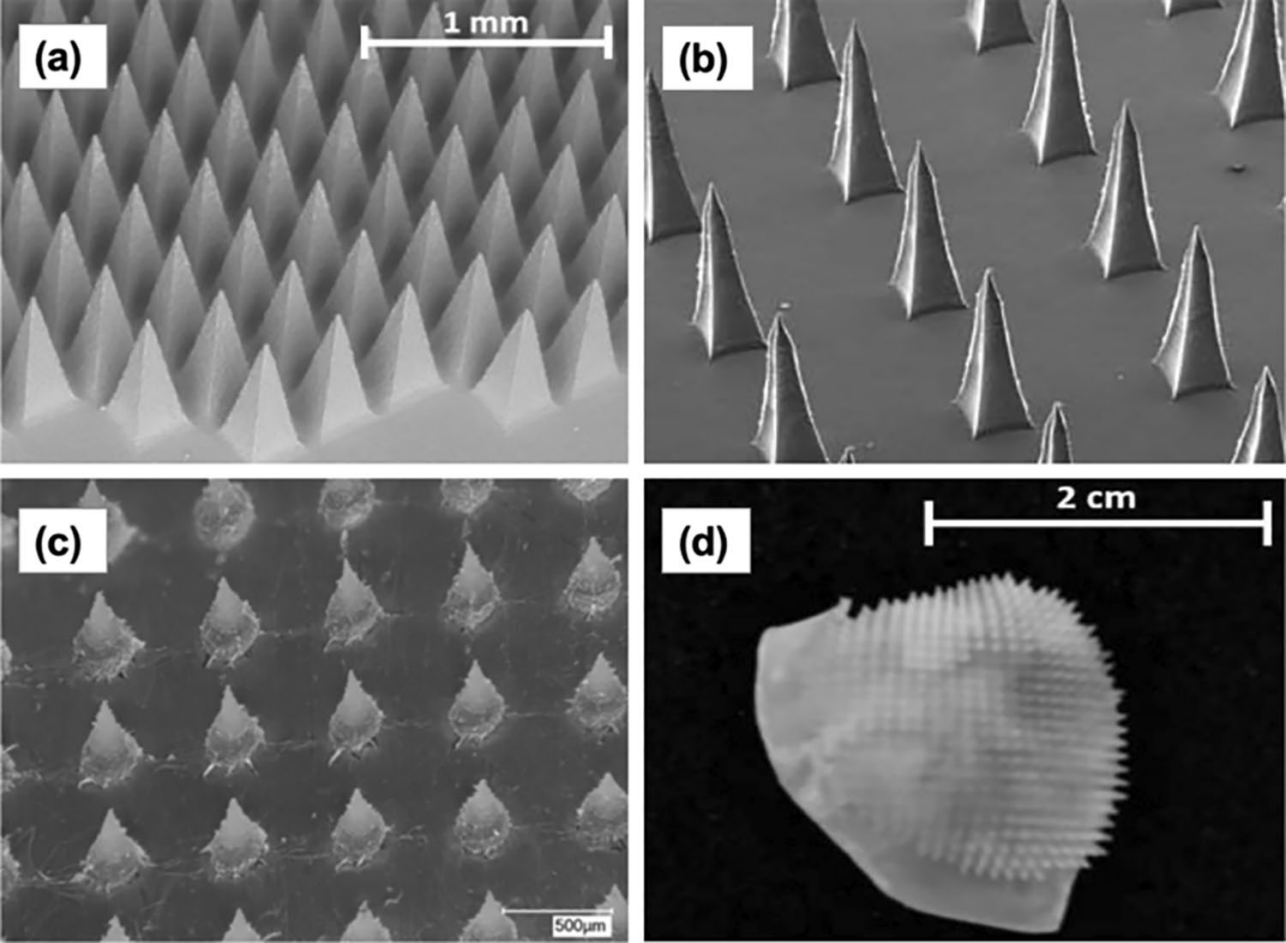
Hydrogel-forming MN made of (**a**) poly(methyl vinyl ether-co-maleic acid) crosslinked with polyethylene glycol [291] (this is an open access article), (**b**) poly(vinyl alcohol) crosslinked-gelatin [289] (this is an open access article) and (**c**) HEMA-crosslinked EGDMA (reprinted with permission from [290] Copyright © 2016, American Chemical Society). (**d**) A swollen hydrogel-forming MN post in vitro permeation study [291] (this is an open access article)

The most recent type of MN developed is hydrogel-forming MNs. Instead of mixing the drugs with polymers, this technology utilises a drug-containing reservoir which is then integrated with blank MN upon application. The difference here is that, after insertion into the skin, the MN can absorb a considerable amount of interstitial fluid and swell in the skin. Then, the drug-containing reservoir dissolves and drug molecules diffuse through the swollen MN conduits into the skin (Fig. 14e). The first hydrogel-forming MN was manufactured from aqueous blends containing poly(methyl vinyl ether-co-maleic anhydride) and poly(ethylene glycol) [232]. Drug-loaded films were integrated with hydrogel-forming MN for delivery of six different drugs, including BSA, insulin, caffeine, theophylline and metronidazole. This invention has become a pioneer of some other studies that have coupled hydrogel-forming MN with different kinds of reservoir, such as lyophilised wafers [284–286], co-solvents [287] and compressed tablets [276]. Some other materials, such as poly(methyl vinyl ether-co-maleic acid)-crosslinked pectin [288], poly(vinyl alcohol) crosslinked-gelatin [289] and 2-hydroxyethyl methacrylate (HEMA)-crosslinked ethylene glycol dimethacrylate (EGDMA) [290], have also been investigated for fabricating hydrogel-forming MN. Figure 19 depicts some representative images of hydrogel-forming MN made of different polymers: (a) poly(methyl vinyl ether-co-maleic acid) crosslinked with polyethylene glycol [291], (b) poly(vinyl alcohol) crosslinked-gelatin [289], (c) HEMA-crosslinked EGDMA [290] and (d) a swollen hydrogel-forming MN post in vitro permeation study [291].

Hydrogel-forming MNs have been used for enhancing transdermal absorption of various active compounds. Donnelly et al. [284] have previously investigated the use of hydrogel-forming MN and lyophilised wafers for transdermal delivery of small and large molecules, namely ibuprofen sodium and ovalbumin, respectively. Similarly, the capability of hydrogel-forming MN in enhancing skin absorption of small molecules has also been reported by Kearney et al*.* [292]. In this study, hydrogel-forming MNs were combined with film reservoirs for delivery of donepezil in a rat model. Subsequently, in 2018, lyophilised wafers integrated with hydrogel-forming MN were successfully employed to improve bioavailability of metformin hydrochloride in a rat model [285]. In terms of protein samples, Courtenay et al. [278, 286] have reported the potential use of hydrogel-forming MN for delivery of macromolecules, such as ovalbumin and bevacizumab, in an in vivo study.

Hydrogel-forming MNs show some benefits when compared to the other type of MNs (solid, coated, hollow and dissolving). Firstly, as the drugs are not a component part of the MN matrix, it is possible to load a greater amount of drug into the associated drug-loaded reservoir than could be loaded into the MN arrays themselves. Consequently, this can lead to increased drug concentrations delivered into the skin. Secondly, following application, the swollen MN are removed intact from the skin; hence, there is no polymer deposition in the skin. Thirdly, hydrogel-forming MN only requires a one-step application process, leading to ease of administration [224]. These advantages have shown that hydrogel-forming MN can be considered as a promising strategy to enhance drug delivery across the skin.

### Materials selection and manufacturing methods of microneedles

Initially, MNs were fabricated using microelectromechanical systems (MEMS) [49]. MEMS is a technology for creating microcomponents which combines mechanical and electrical aspects [293]. This technology was firstly used for manufacturing complex structures on a micron scale, particularly in the integrated circuit industry [294]. MEMS is known by distinctive names in different countries, such as micromachines in Asia or microsystems technology (MST) in Europe [295]. MEMS can be found in many products, such as airbag sensors, data storage, fiber optic networks, inkjet printer heads, projections screen and telecommunication devices [296]. MEMS are employed in different areas of application, including medical devices (implant and biosensor) and bio-MEMS (DNA sequencing instruments and microtitreplates) [297, 298].

**Fig. 20:**
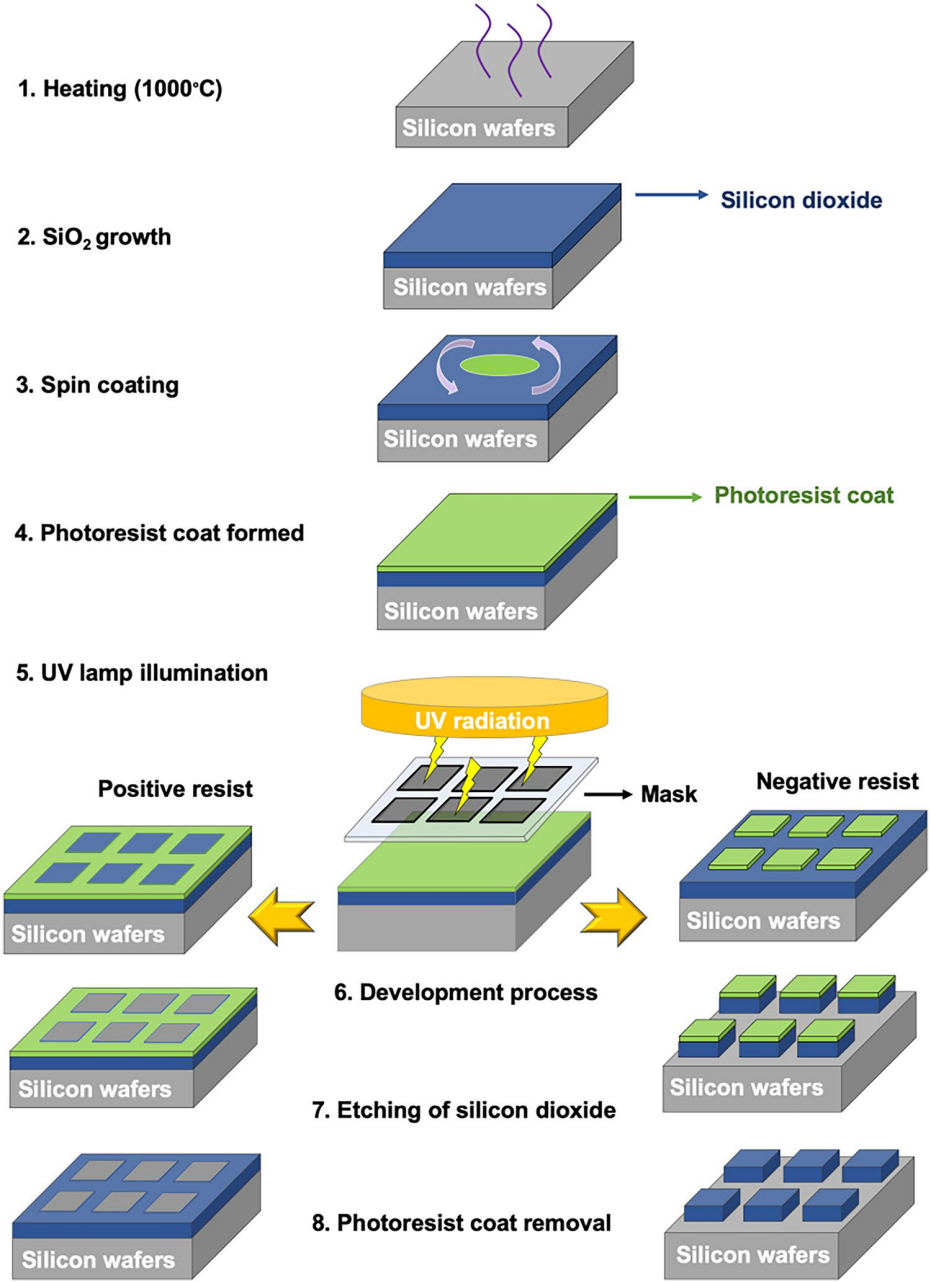
A schematic diagram illustrating all processes involved in photolithography

The earliest form of MEMS is lithography. This technology was invented by Alois Senefelder in 1796 [293]. Lithography (originating from the Greek words meaning ‘writing pattern in a stone’) were primarily used for printing text or artworks on paper or other materials. Then, in 1855, Alphonse Poitevin combined light with lithography based on his photography skill, and this was termed photolithography [299]. In 1955, for the first time, Andrus and Bond used a photoengraving technique (photolithography) for creating patterns on silicon wafers. In 1998, photolithography was then adapted for fabricating silicon MN [228]. Figure 20 summarises the processes involved in the photolithographic technique.

Prior to the main process, it is imperative to ensure that the surface of silicon wafers is clean from dust particles or other organic materials. A solution composed of ammonium hydroxide–hydrogen peroxide–water (1:1:5, v/v) is used for removing organic contaminants and heavy metals [293]. Then, another solution consisting of hydrochloride acid–hydrogen peroxide–water (1:2:8, v/v) is employed for cleaning the surface from magnesium, aluminium and light alkali ions [293].

The first step in photolithography is exposing the silicon wafers to humidified oxygen or steam at high temperature (1000 °C) [49], as displayed in Fig. 20 (step 1). This process will grow a thin layer of silicon dioxide (SiO_2_) on the surface of the silicon wafers, as represented by the blue layer in the second step in Fig. 20. This oxide layer tends to adsorb water molecules from the air and creates silanol groups (Si–OH) which result in poor adhesion of the photoresist materials [293]. Therefore, the wafers are preheated by placing in an oven (200–250 °C) for approximately 30 min to remove water molecules.

Following the growth of silicon dioxide and water removal, an adhesion promoter (hexamethyl disilazane) is added onto the wafer surface before the photosensitive/photoresist material is applied. A spin coating (1500–8000 rpm) process (step 3) is then performed to cover the silicon dioxide surface with the photoresist organic polymer. After the photoresist coat is formed (step 4), the wafers are soft baked at temperatures of 75–100 °C to increase the adherence of each layer [49]. The next step is the UV lamp illumination (150–500 nm) of the photoresist layer through a mask (step 5). Subsequently, the mask pattern will be printed onto the photoresist layer and then the development process takes place.

In the positive resist, the exposed layer will become more soluble in the development solutions due to the weakening of chemical bonds within the resists [49]. Thus, the exposed part will dissolve in the development solution (sodium or potassium hydroxide) [293]. On the other hand, in the negative resist, the exposed part is chemically strengthened and its solubility decreased in the development solution [296]. The reduction of solubility may be caused by the increase of MW of the resist after exposing the UV light or the formation of insoluble products due to the photochemical transformation of the resist [293]. After the pattern is transferred and the development process is complete, an etching process of silicon dioxide is carried out. Etching is a method for removing the selected parts using based on the imaged photoresist after the pattern of the mask is transferred. The most common etching methods are wet etching (using chemical solution) and dry etching (using vapour gas) [296]. Following the etching process, the photoresist layer is then removed.

Besides silicon, other materials have also been explored for manufacturing MN. MN can be made of metals, such as titanium [240], stainless steel [247] and nickel [262]. Several methods are employed for fabricating metal MN, such as electroplating, laser cutting and photochemical etching [300]. Metal MN can also be prepared by assembling stainless steel hypodermic needles on a poly(etheretherketone) mould [241]. Moreover, an infrared laser technique was also used for manufacturing this type of MN [247]. Despite the wide ranges of manufacturing methods and their promising ability in piercing the skin, metal MNs have some disadvantages, such as the possibility of immune-inflammatory responses at the application site and their use may cause an allergic reaction [49]. Thus, different materials were investigated for fabricating MN.

Polymer-based MNs, also known as polymeric MNs, have some benefits compared to the materials mentioned above. For instance, this type of MN is biocompatible, unlike silicon or metal [300]. Furthermore, numerous kinds of polymers have been studied for manufacturing polymeric MN, such as galactose [301], maltose [302], PLGA [231], carboxymethyl cellulose [303], poly(vinyl alcohol) [304], poly(vinyl pyrrolidone) [305], sodium hyaluronate [306], copolymer of methylvinylether and maleic anhydride [307], sodium alginate [308] and poly(acrylic acid) [309]. In addition, polymeric MN can be prepared using cost-effective and simple manufacturing methods, such as micromoulding-based technique [300]. Poly(dimethylsiloxane) (PDMS) or silicone elastomer are the most common materials for making MN moulds [310]. To prepare the PDMS mould, a master structure of the mould is required. This master mould can be made of metal or silicon that is printed using a photolithography method. Specifically, metal master moulds are generally made by cutting from a solid block. However, the master mould made of metal or silicon showed a limitation on the MN height and density [49].

**Fig. 21:**
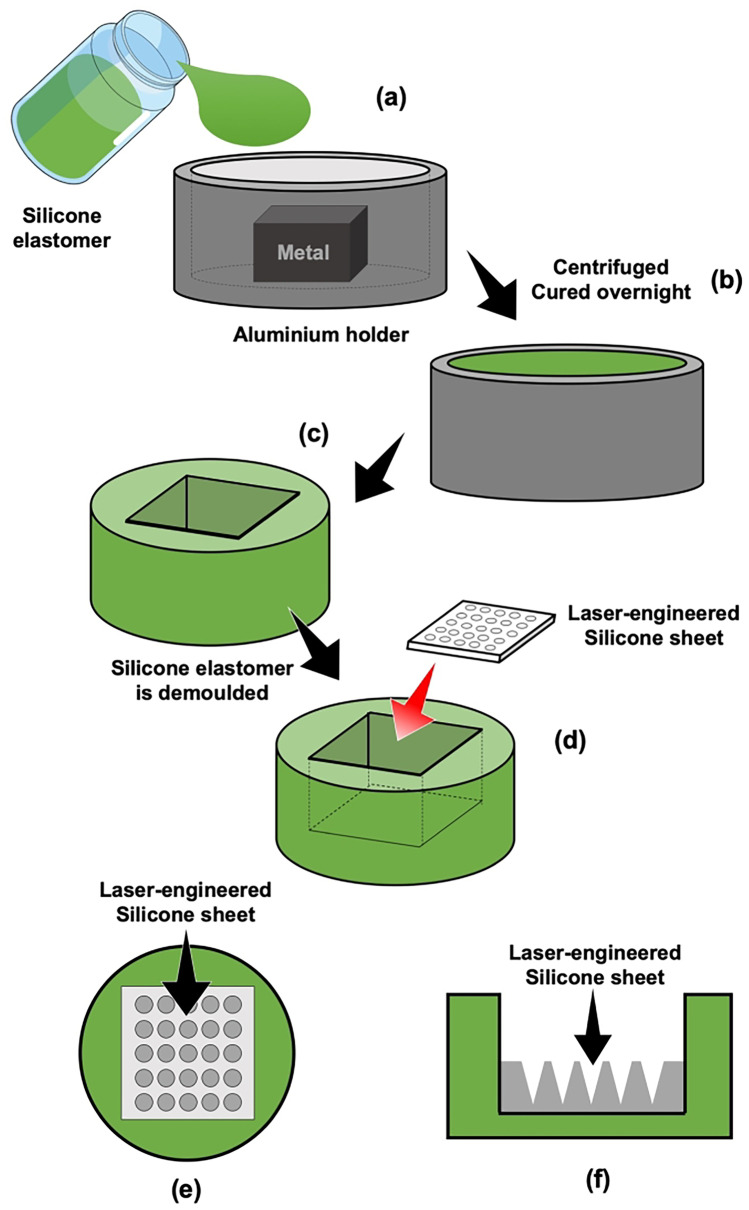
Schematic illustration describing a combination of micromoulding method and laser-based fabrication prior MN manufacturing. (**a**) Silicone elastomer is poured into the aluminium holder with a metal block inside it. (**b**) The aluminium container is filled with silicone elastomer, then this is centrifuged and cured overnight. (**c**) The dry silicone elastomer is demoulded from the aluminium holder. (**d**) A laser-engineered silicone sheet is placed and adhered onto the bottom part of the cast silicone elastomer. (**e**) Aerial and (**f**) cross-sectional view of adhered laser-engineered mould

In order to overcome the problems stated above, Donnelly et al*.* [307] combined the micromoulding method with a laser-engineered technique, as illustrated in Fig. 21. Laser technology utilises monochromatic light which can focus on a tiny spot [49]. Instead of printing the MN structure on the PDMS mould using a metal master mould, laser technology was used to create the MN structures, of relevant height, diameter and spacing, on silicone elastomer sheets. Thus, in this combination method, a laser-engineered silicone sheet is then adhered onto the bottom part of the PDMS or silicone elastomer by crosslinking. This system is then used as the master mould for manufacturing polymeric MN (Fig. 21a–f).

**Fig. 22:**
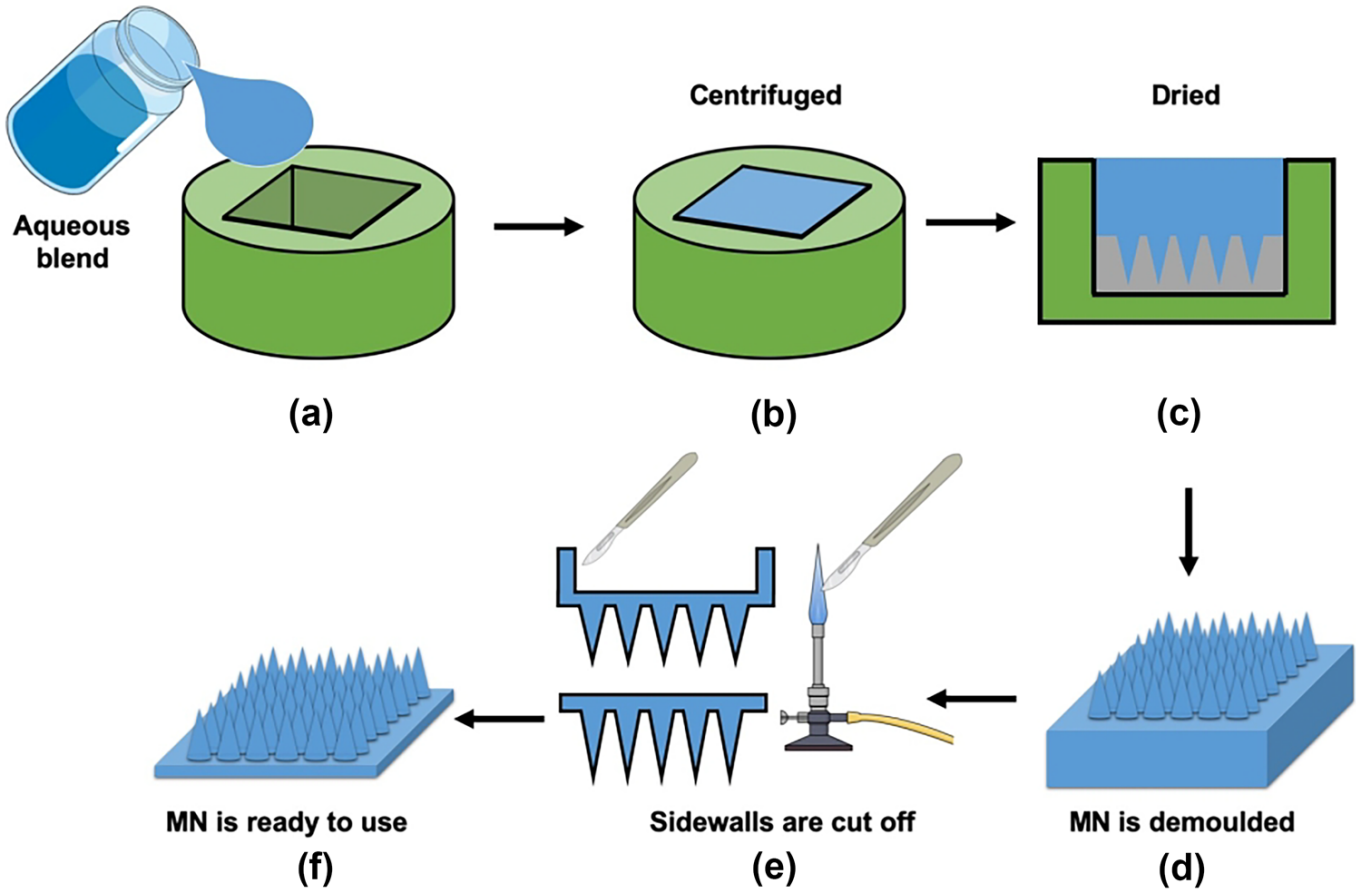
Schematic representation of MN fabrication using micromoulding method which combined with laser-engineered silicone sheet. (**a**) An aqueous blend of polymer is poured onto the laser-engineered silicone sheet inside the green silicone elastomer mould. (**b**) The cast blend is then centrifuged. (**c**) Cross-sectional view of a silicone mould filled with aqueous polymeric blend during the drying process. (**d**) The dry MN is removed from the mould. (**e**) The sidewalls of the MN are cut off using a heated scalpel blade. (**f**) The MN following removal of the sidewalls

To then fabricate MN using these moulds, the selected polymers are dissolved in water to form an aqueous blend. A defined mass of this blend is then poured into the silicone mould. Then, the cast blend is centrifuged to fill the cavities and remove the bubbles during casting process. Following the centrifugation, the cast blend is then dried. The duration of drying is dependent on the type of polymer and composition of the formulation. After the MN are completely dry, they are carefully removed from the silicone mould, and the sidewalls are cut off using a heated scalpel blade, a process that can be operationalised in industrial manufacturing settings. These processes are summarised in Fig. 22a–f.

## Current trend and prospective applications of microneedles

Since this delivery platform can be manufactured using a wide range of materials, scientists are currently intrigued to develop MN for several purposes, such as delivery of corona virus 2019 (Covid-19) vaccines, long acting treatment for human immunodeficiency virus (HIV), contraceptive products and for diagnostic purposes.

MN is one of many strategies which can be applied for enhancing delivery of drug both transdermally and intradermally. The ability of MN to pierce the skin and penetrate into the viable epidermis allows this drug to deliver macromolecules, including vaccines, into the skin. There are many immune cells in the skin, such as Langerhans cells, T cells, macrophages, lymphocytes, mast cells and dendritic cells [35]. The dendritic cells are antigen-presenting cells which function to present the antigens to T cells and stimulate the immune system on the presented antigens [311]. This mechanism is beneficial for promoting immune system and prevents infections caused by viruses.

One virus of note which has emerged since late 2019 is COVID-19 [312]. This virus can cause harmful and deadly infections with mild until critical symptoms [313]. For instance, the number of people tested positive and deaths (with COVID-19 on the death certificate) in the United Kingdom (UK) up to 5 December 2020 was 1,705,971 and 60,916, respectively [314]. In terms of positive cases and deaths globally until 6 December 2020, World Health Organization (WHO) has updated the data in their website and listed 65,870,030 of confirmed cases and 1,523,583 number of deaths [315]. The number of positive cases and death have triggered researchers to develop vaccine for preventing the COVID-19 infections.

With respect to the development of MN for delivery of COVID-19 vaccine, Kim et al. [316] have successfully delivered recombinant coronavirus vaccines in mice. Shin et al*.* [317] have also proposed a degradable MN for COVID-19 vaccine delivery system in their review article. They mentioned that an ideal platform for delivering COVID-19 vaccine should have simple integration system model which are able to be produced widely, not expensive and can be administrated with minimum instruction which reduces supervision [317]. In further work by Abbas et al. [318], the authors have reviewed the benefits of combining MN with nanoparticles for COVID-19 vaccine delivery. Since MN can be manufactured using biomaterials, this delivery platform has been projected as a prospective strategy to address challenges in tackling COVID-19 [319]. Another interesting application of MN has been reported by Chen et al*.* [320]. In their paper, they developed a flexible polymeric MN made of alginate polymer and used this MN as the heads of regular oropharyngeal swabs for improving the efficiency of sampling process during COVID-19 detection. These published sources have emerged the potential of MN for COVID-19 delivery and sampling process to reduce false negative results in COVID-19 detection.

MN technology has been shown to be useful in the delivery of long acting antiretroviral drugs. Long-acting delivery of antiretroviral (ARV) drugs is required for minimising the adverse effect of these drugs. When the drugs are administered orally for a long time, these may cause treatment fatigue which can result in poor patient compliance [271]. Nyaku et al*.* [321] have previously published a summary of promising long acting delivery system for ARV drugs, including nanoformulations, microparticles and implants. Our research group (Donnelly et al.) have been developing a combination of nanoparticle formulations with dissolvable MN for a long acting delivery of ARV drugs. McCrudden et al. [271] have reported the use of dissolving MN for delivery of rilpivirine. They reported that the combination of rilpivirine-containing nanosuspension and dissolving MN could give mean plasma concentration in rats approximately 430 ng/ml at seventh day of treatment. In different study, McCrudden et al. [322] have also found that intravaginal delivery of rilpivirine using dissolving MN was able to give in vivo plasma concentration at approximately 115 ng/ml at the day 56 endpoint. In terms of MN acceptability for treatment of HIV, Moffatt et al*.* [323] has recently published the first paper which provided the insights of patients with HIV, healthcare professionals and members of lay public on the potential of MN for delivery of ARV drugs. Based on their research, it was known that both participants and respondents gave positive response on the MN application for treatment of HIV. Concerning the pharmacokinetic of ARV delivery using MN, Rajoli et al. [324] developed a physiological-based pharmacokinetic model for estimating the optimum dose regimen and release rate of MN containing cabotegravir and rilpivirine. Each of the studies mentioned outlines in detail a series of positive aspects for patients of MN delivery of ARV drugs. To maximise the use of MN for HIV treatment, it is imperative to give prior knowledge and education to the patients and healthcare professionals about the safety and the clinical applications of MN [323].

With respect to long-acting delivery of drugs using MN, this potential has been also used for delivery of contraceptive agents [325]. As previously discussed, the marketed transdermal products were mostly aimed to contraceptive agent delivery, such as estradiol, levonorgestrel and ethynyl estradiol. Transdermal delivery systems are used for minimising the drawbacks of some other delivery systems. For instances, transdermal delivery systems may overcome the associated drawbacks of oral dosage forms (not a long-acting system and poor patient adherence), injection (due to their invasiveness, pain experience and the need of personnel experts for administration) and implants (more invasive than injection and the requirement of trained healthcare professionals) [326–328]. Nevertheless, transdermal patches themselves have some associated drawbacks, such as the limitation of drugs to permeate across SC because this delivery system relies on passive diffusion [7]. These problems documented above may be overcome by using MN. MN technology can provide more benefits compared to conventional transdermal patches as they can penetrate the skin’s SC. MN can also be used for intradermal delivery of contraceptive agents providing long lasting effects once the drug has been deposited in the skin [7].

Published studies have demonstrated that MNs have much potential for delivery of long-acting contraceptives. Yavuz et al. [329] have investigated the silk fibroin-based MN for levonorgestrel sustained release. They found that MN containing levonorgestrel was able to deliver the drug up to 100 days. However, when the drug was formulated into microparticles before they were cast into MN, such system could deliver levonorgestrel for more than 1 year. A different type of MN was developed by Li et al. [327]. In their study, levonorgestrel-containing needles were attached onto effervescent MN patch which was able to leave the needles in the skin upon insertion. This system is easily administrated and can release the drug slowly up to more than 1 month. Dissolving MNs were also reported for delivery of different contraceptive hormones, such as etonogestrel [330]. Etonogestrel was previously formulated into microcrystal particles prior to MN casting. He et al. [330] have observed that dissolving MN could give similar in vivo bioavailability of etonogestrel when compared to intradermal injections. They proposed that MNs are prospective systems for sustained delivery of etonogestrel and an option to reduce the invasiveness of intradermal injection. A different research published by Li et al. [331] explored the use of 2-layer MN for delivery of levonorgestrel in rats. This rapidly separable MN could provide sustained delivery of levonorgestrel up to 60 days. Brunie et al. [332] reported a qualitative study on the acceptability of contraceptive MN. The investigation was conducted by an in-depth interview with women in India and Nigeria. They concluded that MN can be a promising approach for contraceptive purposes, however some aspects should be considered, including side effects, effectiveness and pricing of the MN. By altering and modifying the number of needles, patch size and the amount of drug formulated in MN, this technology could be a viable alternative option for delivering long-acting contraceptive hormones for women [325].

Besides the delivery function, MNs have also been investigated for therapeutic drug monitoring and bio-sensing. This technology was considered as a minimally invasive monitoring method because it utilises the micron-sized needles for taking the biological samples, such as skin interstitial fluid (ISF) or blood [333]. MNs are used for extracting skin ISF or blood samples from the skin [334], and an appropriate extraction method is employed prior analysis. For example, Rawson et al*.* [335] have published a paper about monitoring of phenoxymethylpenicillin concentration using hollow MN in healthy human volunteers. Moreover, Ito et al*.* [336] used dissolving MN for monitoring the concentration of vancomycin in dermal ISF. Hydrogel-forming MNs have also been reported for drug monitoring purposes, as previously reported by Caffarel-Salvador et al*.* [337], who used this type of MN for extracting and quantifying drug substances and glucose from skin.

The applications of MN which have been documented above have indicated that MN continue to be a promising technology for many purposes. It is not surprising, therefore, the World Economic Forum listed MN as one of ‘Top 10 Emerging Technology of 2020’ [338]. However, they have also mentioned in their report that further researches are required for evaluating the factors which can affect the delivery and effectiveness of MN [338]. Moreover, for future consideration, there are some concerns which have to be standardised, such as the application process and patient’s prior knowledge and understanding of new MN technologies. Additionally, a programme of clinician education might be required for the clinical application of MN.

## Conclusion

Transdermal delivery systems have been developed as a solution for overcoming problems associated with oral or injection dosage forms. There are many enhancement strategies that can be applied for improving transdermal delivery systems. Passive transdermal drug delivery technologies have been employed in the majority of marketed products. Nevertheless, this delivery system is limited to the small MW and hydrophobic drugs. The combination of energy driven methods, such as iontophoresis, with conventional transdermal patch (Zecuity^®^) has given an alternative option for improving passive transdermal delivery system. Even though this approach sounds promising, this technology still cannot be used for delivering the macromolecules. Recent studies have shown that active method by piercing skin’s SC, namely MN, is one of promising approaches for delivery of various kind of drugs. By combining MN with conventional transdermal patches, it is more possible to deliver peptide/protein-based drugs via transdermal route. The applications of MN have been increasing since the first type of this delivery platform was developed in 1998. Published papers have also proved that MN can be used for improving delivery of drugs. Recently, MN have been successfully reported for many purposes, such as for delivering COVID-19 vaccines, long-acting delivery for HIV treatment and contraceptive hormones, and therapeutic drug monitoring. These have shown that MN technology have been a prospective strategy for improving transdermal delivery system. Furthermore, this delivery platform has been listed as one of ‘Top 10 Emerging Technologies of 2020’ by World Economic Forum. This endorsement by the World economic forum bolsters support for the rapid commercialisation of MN products currently under regulatory review and development. It is important that regulatory oversight is comprehensive for this emerging technology, and that all aspects of commercialisation are fully addressed to ensure MN technology can have a positive impact on patients and clinicians across the entire medial field. For instance, repeat bioequivalence studies must be conducted to ensure the safety, efficacy and validity of clinical phase data. Additionally, it is important that MN production and industrial scale-up is considered fully. This concern is often associated with the aseptic manufacture and large-scale fabrication processes. In addition, it is not surprising that advanced technology and hi-tech apparatus will be needed for the necessary quality assurance procedures. Consequently, this high-cost process will influence the price of the final product. Furthermore, before such a product can be considered for use in clinical settings, the relevant regulatory authorities must provide guidance on appropriate manufacturing conditions so that MN can be produced in a cost-effective manner.
